# Supplementary material for: Mitochondrial CaMKII causes adverse metabolic reprogramming and dilated cardiomyopathy
Source: Nat Commun. 2020 Sep 4;11:4416. doi: 10.1038/s41467-020-18165-6 (PMC7473864; doi:10.1038/s41467-020-18165-6)
Supplement: Supplementary file 1 — Supplementary Information [file 41467_2020_18165_MOESM1_ESM.pdf]

Supplementary Information

Mitochondrial CaMKII causes adverse metabolic reprogramming and dilated cardiomyopathy

Luczak, et al

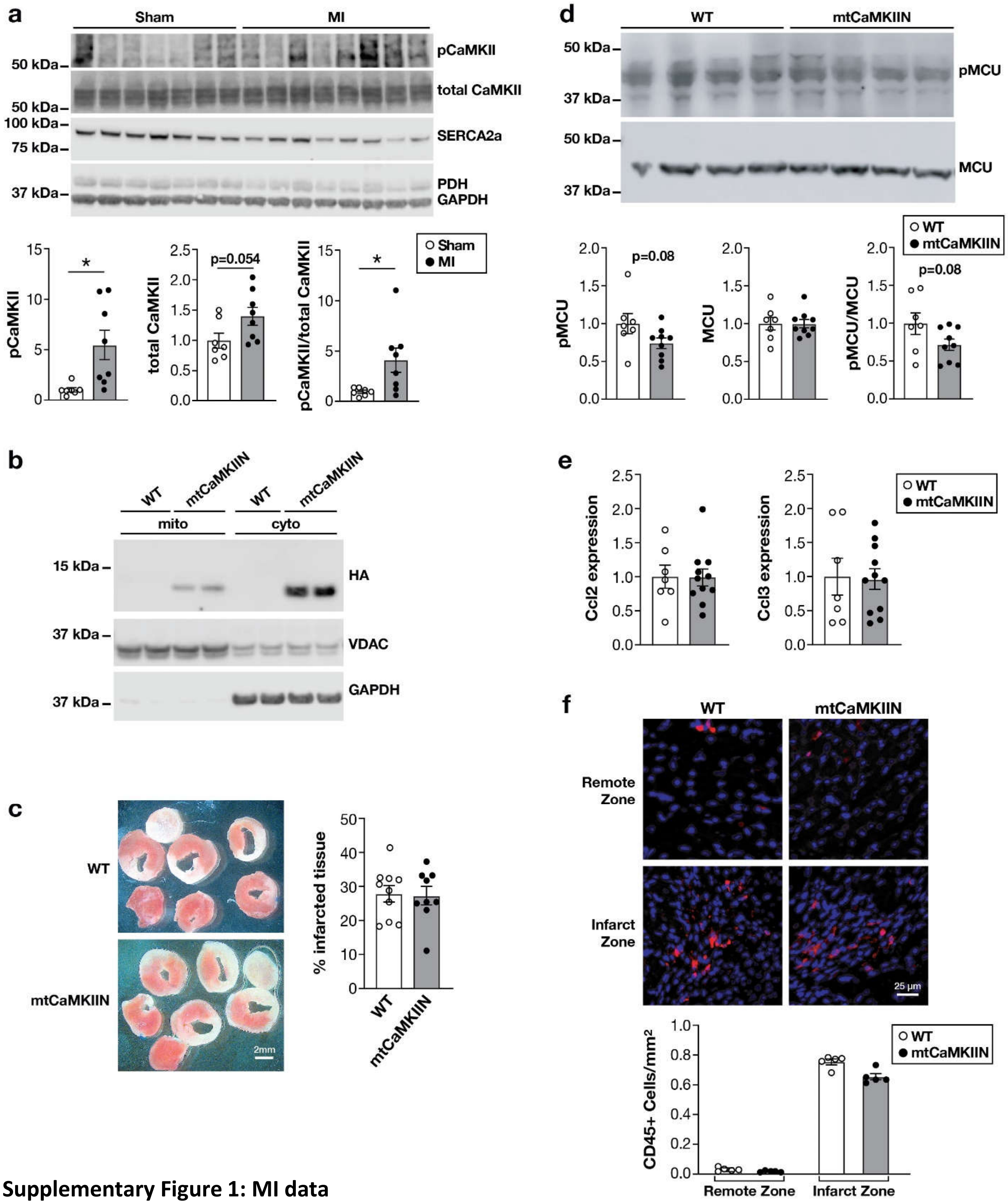

Supplementary Figure 1: MI data

a Western blot of cytosolic lysates and summary data for phosphorylated CaMKII and total CaMKII normalized to coomassie staining in mitochondria from sham (n=7) and MI (n=8) hearts. Blots for cellular compartment markers also included: SERCA2a (SR membrane), PDH (mitochondrial matrix), GAPDH (cytosol). **b** Western blot for HA-CaMKIIN, VDAC1 and GAPDH in cytoplasm and mitochondria fractions from WT (n=2) and mtCaMKIIN (n=2) hearts. **c** Representative images and quantification of of TTC staining in WT (n=10) and mtCaMKIIN (n=9) hearts 24 hours after MI surgery. **d** Representative western blot and summary data of pMCU and MCU normalized to Coomassie staining in mitochondrial lysates from WT (n=7) and mtCaMKIIN (n=9) 1 week after MI. **e** qPCR quantification of Ccl2 and Ccl3 mRNA normalized to Hprt in WT (n=7) and mtCaMKIIN (n=11) hearts 1 week after MI. **f** Representative immunofluorescence images and summary data for CD45+ cells in heart sections of WT (n=5 hearts) and mtCaMKIIN (n=5 hearts) hearts 1 week after MI. Data are represented as mean ± SEM, significance was determined using two-tailed Student's t test. *P<0.05. Source data, including exact p values, are provided as a Source Data file.

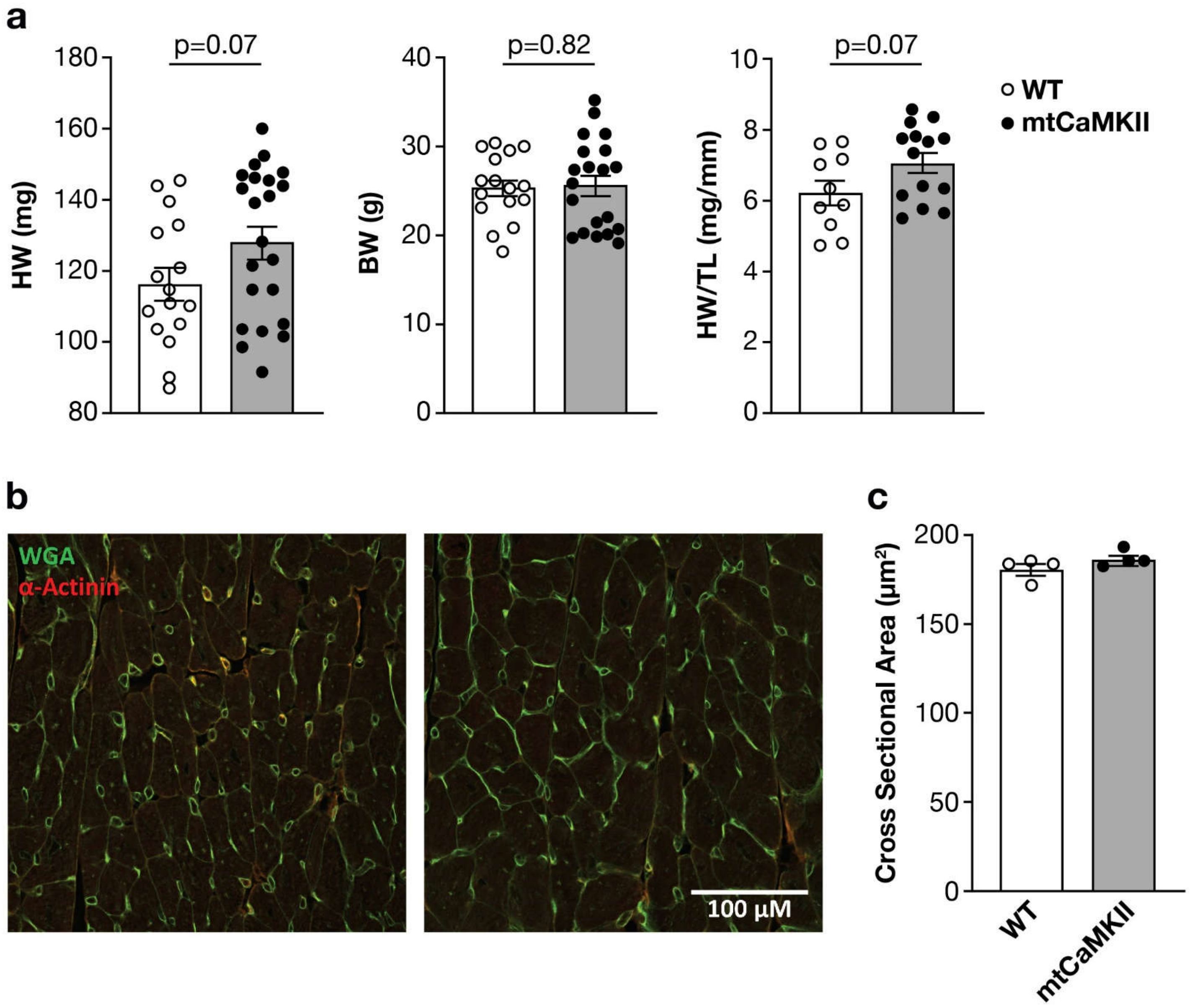

Supplementary Figure 2: mtCaMKII mouse data

a Summary data for HW (WT n=16, mtCaMKII n=22), BW (WT n=16, mtCaMKII n=22) and HW/TL (WT n=10, mtCaMKII n=14) measurements for WT and mtCaMKII mice. **b** Representative images and **c** summary data for quantification of cardiomyocyte cross sectional area in WT (n=4 hearts) and mtCaMKII (n=5 hearts) heart sections. Data are represented as mean ± SEM, significance was determined using two-tailed Student's t test. Source data are provided as a Source Data file.

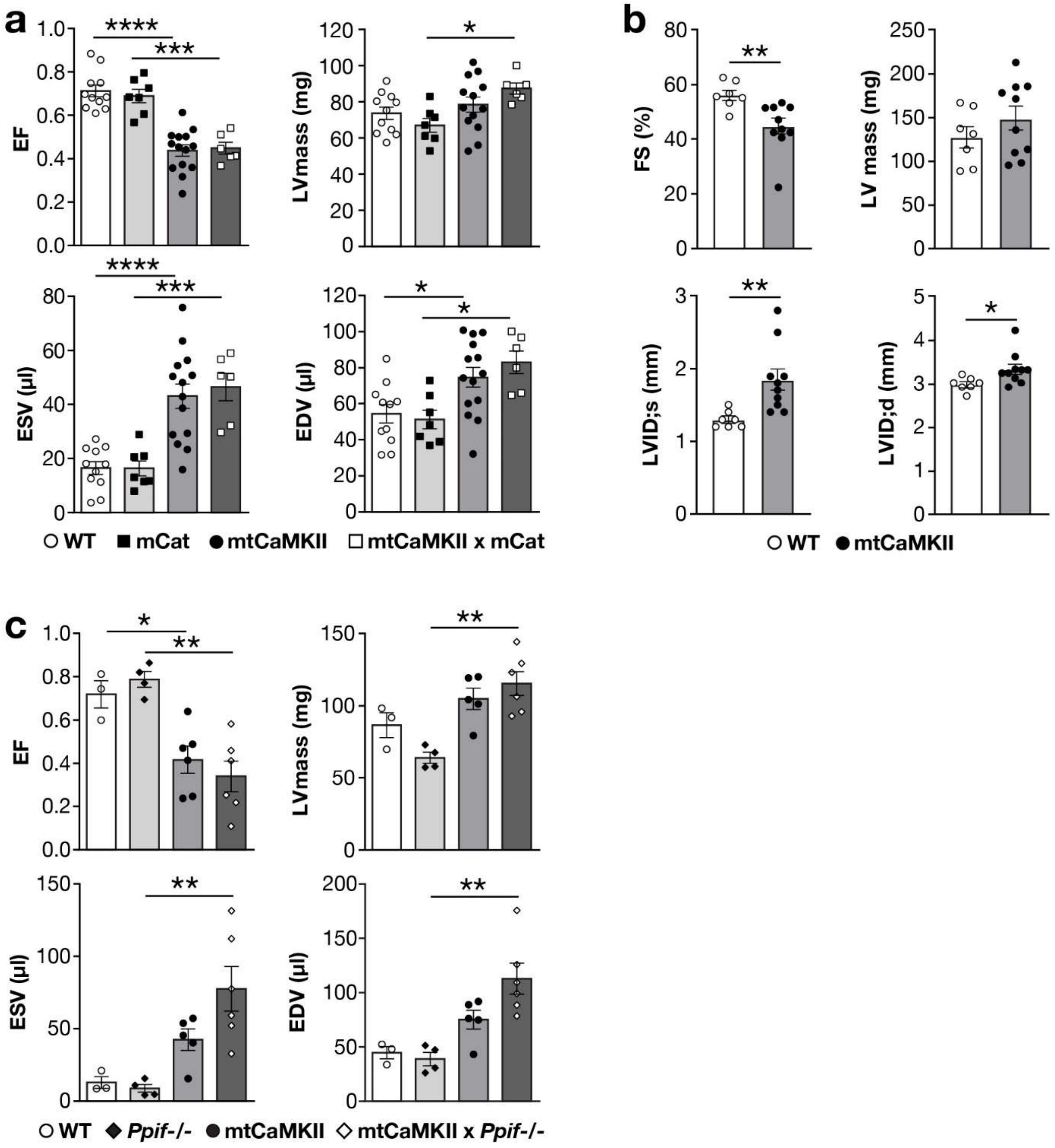

Supplementary Figure 3: Genetic crosses do not rescue mtCaMKII dilated heart phenotype

a Summary data from echocardiographic measurements in WT (n=11), mitochondrial-targeted catalase over-expressing (mCat) (n=7), mtCaMKII (n=14), and mtCaMKII x mCat interbred (n=6) mice, **b** WT CD1 (n=7) and mtCaMKII CD1 (n=10), and **c** WT (n=3), *Ppif*$^{-/-}$ (n=4), mtCaMKII (n=6), and mtCaMKII x *Ppif*$^{-/-}$ interbred (n=6) mice. Data are represented as mean ± SEM, significance was determined using two-tailed Student's t test or 1 way ANOVA with Tukey's multiple comparison's test. ****P<0.0001, ***P<0.001, **P<0.01, *P<0.05. Source data, including exact p values, are provided as a Source Data file.

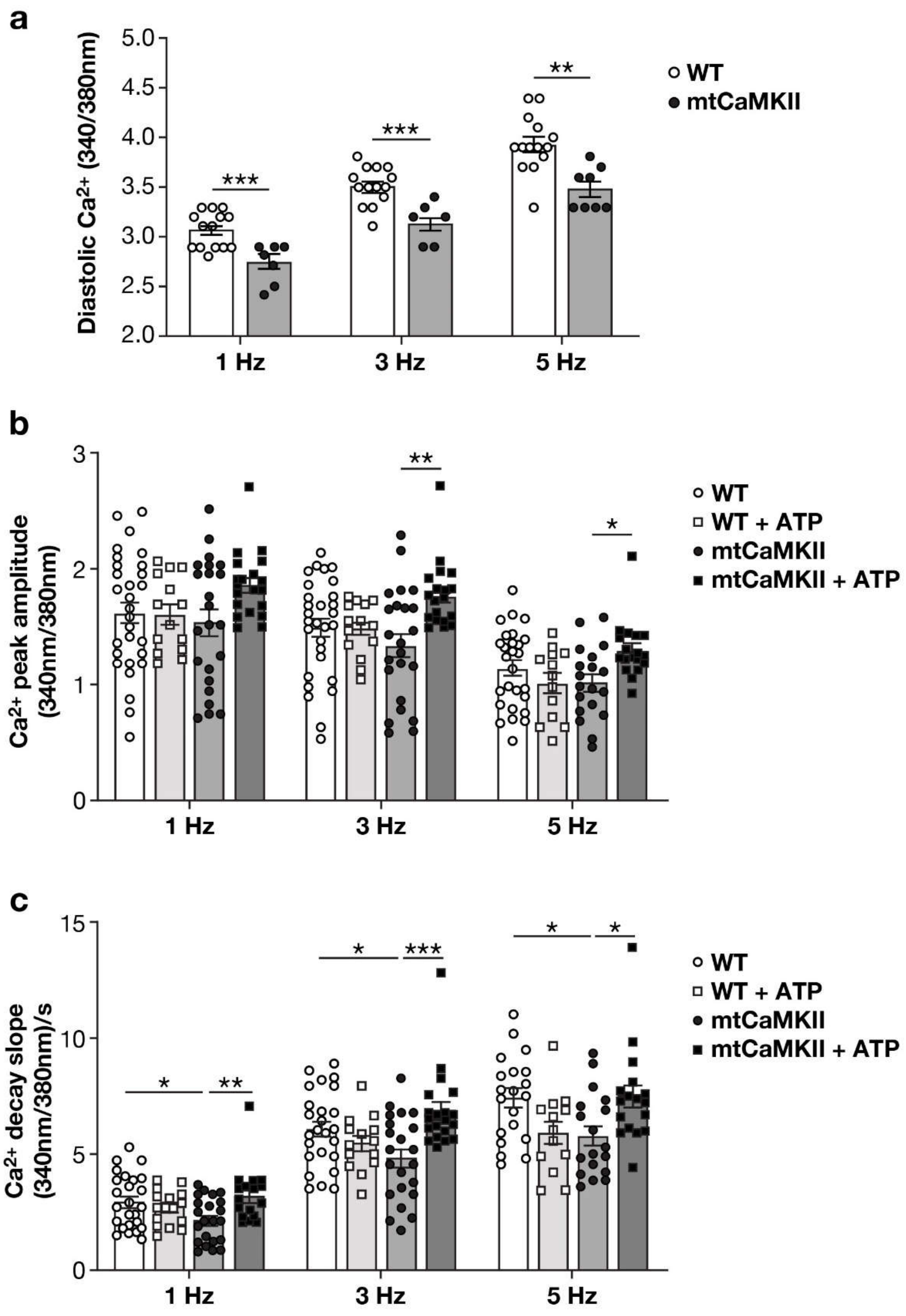

Supplementary Figure 4: [Ca^{2+}] in myocytes

a Summary data for diastolic [Ca^{2+}] measurements made with Fura-2–loaded ventricular myocytes isolated from WT (n=14 cells from 2 hearts) and mtCaMKII (n=8 cells from 2 hearts) hearts and field stimulated (1, 3, and 5 Hz). Myocyte cytoplasm was not perfused with a CaMKII inhibitory peptide (AIP) as in Figs 3e-f and 4h. Summary data for **b** Ca^{2+} peak amplitude and **c** decay slope from isolated ventricular myocytes stimulated at 1, 3 (WT n=25; WT+ATP n=14; mtCaMKII n=22; mtCaMKII+ATP n=20), or 5 Hz (WT n=20; WT+ATP n=13; mtCaMKII n=18; mtCaMKII+ATP n=18) (ventricular myocytes isolated from 2 hearts for WT cells and 3 hearts for mtCaMKII cells). Data are represented as mean ± SEM, significance was determined using a two-tailed Student's t test, or 1 way ANOVA with Tukey's multiple comparison's test. ***P<0.001, **P<0.01, *P<0.05. Source data, including exact p values, are provided as a Source Data file.

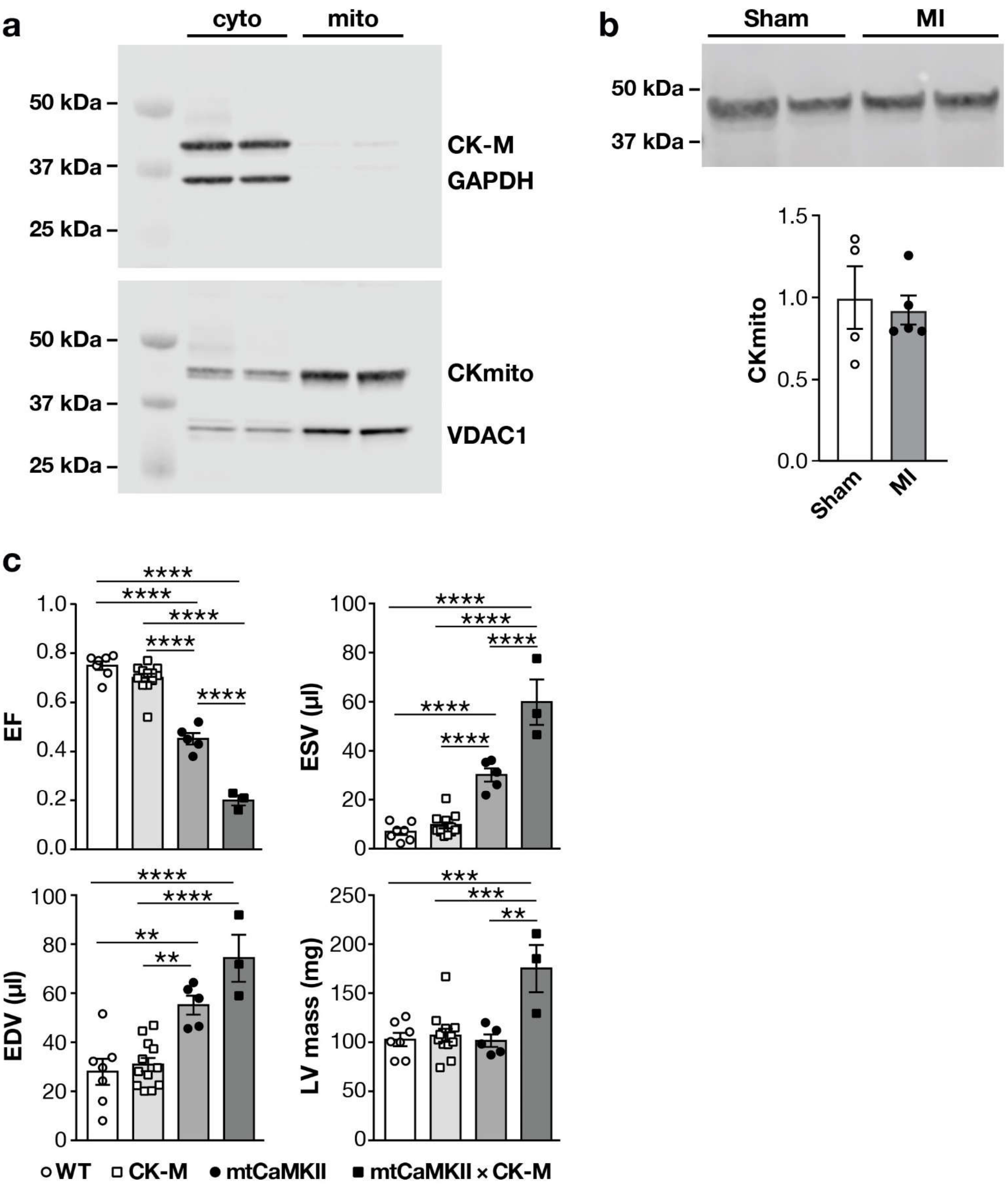

Supplementary Figure 5: CK expression in transgenic and MI hearts

a Western blot for CKmito and CK-M of cytosolic and mitochondrial fractions from WT mouse hearts. This experiment was repeated independently twice with similar results. **b** Representative western blot and summary data for Ckmito in sham (n=4) and MI (n=5) hearts. **c** Summarized echocardiographic measurements from WT (n=7), CK-M (n=12), mtCaMKII (n=5), and mtCaMKII x CK-M (n=3) mice. Data are represented as mean ± SEM, significance was determined using 1 way ANOVA with Tukey's multiple comparison's test. ****P<0.0001, ***P<0.001, **P<0.01. Source data, including exact p values, are provided as a Source Data file.

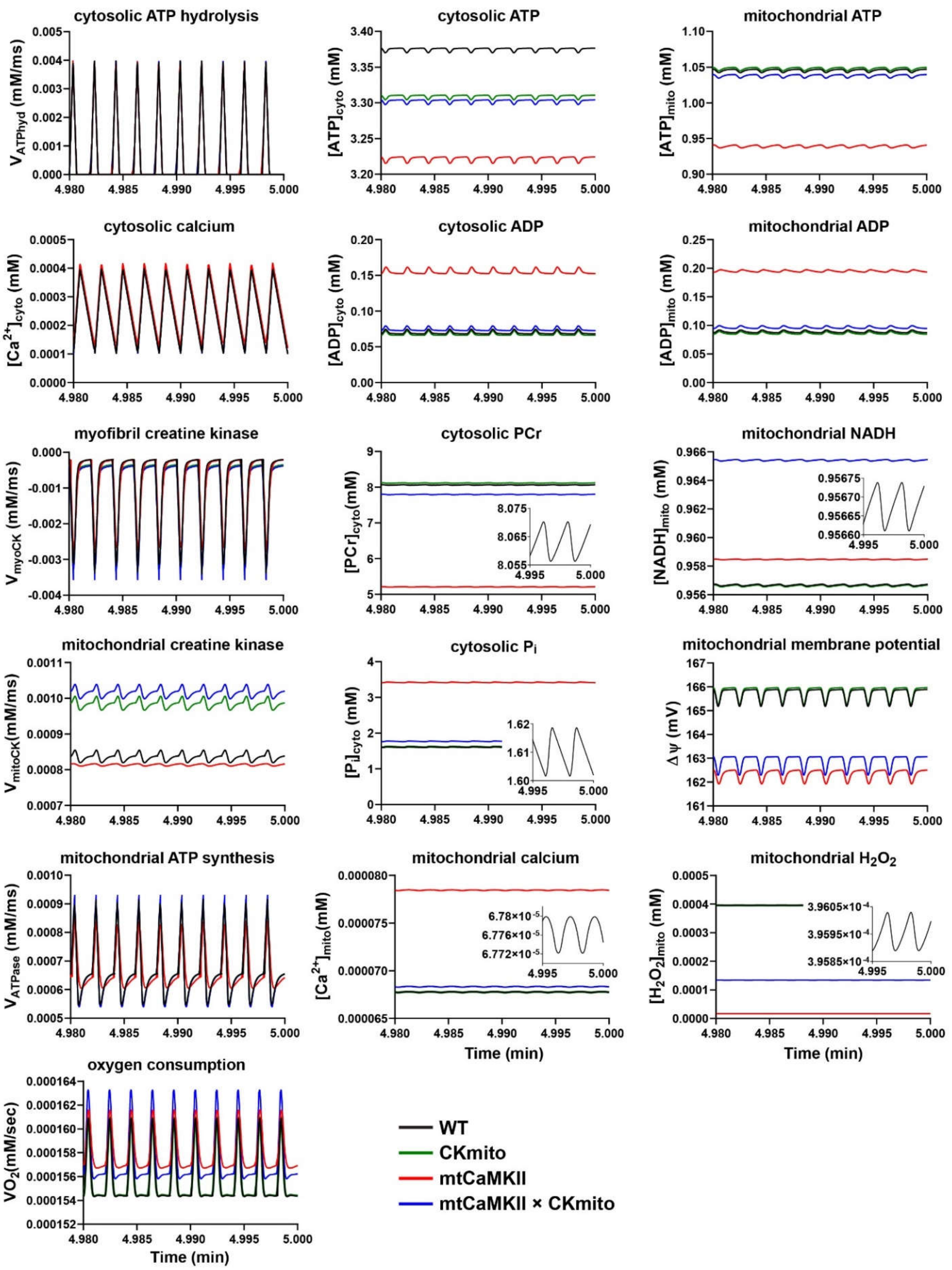

Supplementary Figure 6: Computational Model Outputs

ATP hydrolysis and cytosolic calcium were simulated using pulsatile functions to simulate cardiac contraction. Mitochondrial creatine kinase flux, myocardial creatine kinase flux, mitochondrial ATP synthesis rate, and oxygen consumption as well as ATP, ADP, PCr, Pi levels, and mitochondrial NADH, membrane potential, H_2O_2 and Ca^{2+} were compared in WT, CKmito, mtCaMKII, and mtCaMKII x CKmito interbred conditions after 5 minutes of simulation. Insets provide zoomed in scale to show small oscillations. Only WT is shown in the inset for clarity.

Pathway		Phosphoproteins		
Metabolism	TCA Cycle	ACON	CISY	MDHM
		ODO1	ODO2	ODPA
		ODPX	SUCB1	
	Electron transport/ Oxidative Phosphorylation	ATP5H (CV)	COX41 (CIV)	NDUFB11 (CI)
		UQCC2 (CIII)		
	Fatty Acid Oxidation	ACADV	ACSL1	ECI1
		ETFA		
	Other Metabolic	AATM	ADAS	ARK72
		GCSH	KCRS	MUTA
Transcription/Translation		EFTS	NUCKS	PERM1
		RM01	RT02	RT23
		SLIRP	SYIM	
Oxidative Stress		MSRB2	PRDX3	PRDX5
Import		TIM44	TOM70	
Fission/Fusion		MFR1L		
Unknown		CQ059	OCAD1	

Supplementary Table 1: CaMKII has diverse mitochondrial targets

Proteins identified by LC/MS analysis to have increased phosphorylation in mtCaMKII compared to WT mitochondria. Peptides were considered to be significantly more phosphorylated with a fold change of ≥ 2. Proteins were classified by function.

Parameters	WT	CKmito	mtCaMKII	mtCaMKII x CKmito	Source
**Complex I expression/activity**	100%	100%	45%	70%	Fig 5E and H
**CKmito expression/activity**	100%	120%	80%	120%	Fig 4E and F
**Cytosolic ATP hydrolysis**	100%	100%	100%	100%	
**TCA cycle enzyme activity (IDH, α-KGDH, FH, MDH)**	100%	100%	120%	120%	Fig 6B
**Complex II expression/activity**	100%	100%	130%	130%	Fig 5E
**Diastolic cytosolic calcium**	100%	100%	120%	100%	Fig 3E and 4H
**Peak cytosolic calcium**	100%	100%	105%	100%	Fig 3E

Supplementary Table 2: Experimental data used for computational modeling

Summary of the parameters used in the computational model that were taken from the experimental data for WT, CKmito, mtCaMKII, and mtCaMKII x CKmito.

Supplementary Methods

TTC staining and MI sizing
At the end of 24 hours post-MI, mice were sacrificed, the hearts quickly excised, and both atria and right ventricular free wall were removed. Left ventricular tissue was weighed and wrapped with cling film and then frozen at -20°C for 45 min, and then sliced into 6-7 pieces of 1.0 mm thick sections perpendicular to the long axis of the heart. The sections were incubated individually using a 24-well culture plate in 1% TTC in phosphate-buffered saline at pH 7.4 at 37°C for 10 min), and then digitally photographed. For infarct size at 24 hours post-MI, TTC-stained area, and TTC-negative staining area (infarct myocardium) were measured using ImageJ. Myocardial infarct size was expressed as a percentage of the total LV area.

Quantitative PCR
Total RNA was prepared using Trizol reagent. iScript™ Reverse Transcription Supermix (Bio-Rad) was used to generate cDNA from RNA. Validated PrimePCR primers (Bio-Rad) (Ccl2 qMmuCED0048300, Ccl3 qMmuCED0044190) and SsoAdvanced™ Universal SYBR® Green Supermix (Bio-Rad) were used for qPCR on CFX Connect™ Real-Time PCR Detection System (Bio-Rad). Transcript levels were quantified by the ΔΔCt method.

Immunofluorescence
In brief, hearts were fixed in 4% PFA and embedded in OCT freezing compound (Fisher Scientific, Waltham MA, USA). 10 µm cardiac two chamber view sections were cut using a Microm HM 550 cryostat. Sections were post-fixed with 4% paraformaldehyde and permeabilized using 0.1% Triton-X in PBS. Sections were washed two times for 5 minutes with 1x HBSS. Sections were incubated with anti-CD45 (Thermo Fisher, Waltham, MA, USA) (#14-0451-82 at 1:50) according to previously published methodology [1]. Samples were subsequently incubated with Alexafluor 555 at 1:1000 (Thermo Fisher A21434). At least 3-5 fields of view per infarct and remote zone were assessed from n=5 hearts per genotype. Images were acquired in 0.63 µm Z-stacks using an Olympus IX83 microscope, 60x/1.42NA PLAPON objective. CD45+, nucleated cells were counted and normalized number of cells/area (mm^2).

Myocyte cross-sectional area
In brief, hearts were prepared as above. Sections were incubated with 5 µg/mL in Wheat Germ Agglutinin, Alexa Fluor® 488 conjugate (Molecular Probes, Eugene, OR, USA) for 20 minutes. Samples were washed two times for 5 minutes with 1x HBSS and mounted with Prolong Diamond Anti-Fade Reagent (Molecular Probes, Eugene, OR, USA). Ten fields of view were acquired from each animal. At least four animals were processed per genotype. 0.63 µm Z-stacks were collected using an Olympus IX70 microscope, 40x/0.75NA UPLFLN-PH objective, and deconvolved using constrained iterative deconvolution software in the cellSens software suite. Average projections for each field of view were calculated and analyzed using cellProfiler (Cell Profiler Inc. Boston, MA, USA) according to previously published methods to determine cell size.

Computational Model Validation

The model was constructed by integrating two computational models: Kongas and van Beek, 2007 creatine kinase model [2] and Gauthier et al, 2013 cardiac bioenergetic model [3]. The model parameters were directly taken from these two models unless stated otherwise. The metabolite steady-state levels in the control group were matched to the original model ranges as model validation. Simulation codes are available at the URL: https://gitlab.com/MitoModel/mtCaMKII.git. Experimental data for validation is as follows:

1. Mouse heart rate is around 500 bpm (400-600), which is approximated to 120 ms/beat.
2. CKmito mice [4] :

	WT	CKmito	source
CKmito expression	1	1.7	Gupta et al, (2012) Fig1
CK activity	10 IU/mg protein	16 IU/mg protein	Gupta et al, (2012) Fig1
Total CK flux	4 μmol/g/s	12 μmol/g/s	Gupta et al, (2012) Fig4
PCr/ATP	1.8	1.9	Gupta et al, (2012) Fig4
ATP	4 μmol/g wet weight	4 μmol/g wet weight	Gupta et al, (2012) Fig4
pseudo-first-order forward rate constant, kf	$0.5s^{-1}$	$1.5s^{-1}$	Gupta et al, (2012) Fig4
Total CK flux	3 μmol/g/s or 0.0075 mM/ms *	4.5 μmol/g/s	Gupta et al, (2012) Fig8
PCr/ATP	1.9	1.9	Gupta et al, (2012) Fig8
ATP	4.8 μmol/g	4.3 μmol/g	Gupta et al, (2012) Fig8
PCr	9.8 μmol/g	8.5 μmol/g	Gupta et al, (2012) Fig8

* For CK Flux: CKmyo(MMCK) is about 70% of total CK flux (~0.005 mM/ms) and CKmito(MiCK) is 30% of total flux (~0.0025 mM/ms). Total CK flux is about 5 to 10 times of ATP hydrolysis rate.

3. ATP hydrolysis rate and oxygen consumption rate:

- In rabbit heart [5] (135 to 220 beats/min in isolated rabbit hearts), a change in myocardial oxygen consumption from 22.0 to 28.4 µmol·g /dry mass/min corresponded with an estimated ATP hydrolysis from 487 to 628 µmol/l cell water/s.
- In Hettling et al [6], temporal buffering capabilities of the CK isoforms were tested in silico with high peaks of ATP hydrolysis (3750 µM*s−1) in myofibrils.

4. PCr/ATP ratio, PCr and ATP concentrations (MRI, MRS in vivo data in this manuscript):

	**WT**	**mtCaMKII**	**source**
PCr/ATP ratio	2.05	1.66	
PCr	9.55 µMol/gram of wet weight or 23 mM*	5.35 µMol/gram of wet weight or 13.3 mM*	Fig. 4g
ATP	4.66 µMol/gram of wet weight or 11.6 mM*	3.27 µMol/gram of wet weight or 8.1 mM*	Fig. 4g

* It is assumed 1g wet weight tissues corresponds to 0.4ml intracellular water (Vinnakota et al. 2004, Table 5) [7].

Ordinary differential equations used are as follows:

Mitochondrial ions:

$$(1)\quad \frac{d[\mathrm{Ca}^{2+}]_m}{dt} = \delta_{Ca}(J_{uni} - J_{NCLX})$$

$$(2)\quad \frac{d[\mathrm{Na}^{+}]_m}{dt} = J_{NCLX} - J_{NaH}$$

$$(3)\quad \frac{d[\mathrm{H}^{+}]_m}{dt} = \delta_{H}(-J_{Hres} + J_{Hu} + V_{NaH} + J_{PiC} + J_{Hleak})$$

$$(4)\quad \frac{d[\mathrm{Pi}]_m}{dt} = -J_{F1Fo} + J_{PiC} - J_{SL}$$

$$(5)\quad C_m\frac{d\Delta\Psi_m}{dt} = J_{Hres} - J_{Hu} - J_{ANT} - J_{Hleak} - J_{NCLX} - J_{uni} - J_{IMAC}$$

Citric acid cycle:

$$(6)\quad \frac{d[\mathrm{ISOC}]}{dt} = J_{ACO} - J_{IDH3} - J_{IDH2}$$

$$(7)\quad \frac{d[\alpha\mathrm{KG}]}{dt} = J_{IDH3} + J_{IDH2} - J_{KGDH} + J_{AAT}$$

$$(8)\quad \frac{d[\mathrm{SCoA}]}{dt} = J_{KGDH} - J_{SL}$$

$$(9)\quad \frac{d[\mathrm{SUC}]}{dt} = J_{SL} - J_{SDH}$$

$$(10)\quad \frac{d[\mathrm{FUM}]}{dt} = J_{SDH} - J_{FH}$$

$$(11)\quad \frac{d[\mathrm{MAL}]}{dt} = J_{FH} - J_{MDH}$$

$$(12)\quad \frac{d[\mathrm{OAA}]}{dt} = J_{MDH} - J_{CS} - J_{AAT}$$

$$(13)\quad \frac{d[\mathrm{NADH}]_m}{dt} = -J_{C1} + J_{IDH} + J_{KGDH} + J_{MDH} - J_{THD}$$

ROS transport and scavenging:

$$(14)\quad \frac{d[\mathrm{NADPH}]_m}{dt} = J_{IDH2} + J_{THD} - 0.5J_{GR,m} - J_{TxR,m}$$

$$(15)\quad \frac{d[O_2^{\cdot-}]_m}{dt} = J_{ROS,m} - J_{SOD,m} - J_{ROS}^{Tr}$$

$$(16)\quad \frac{d[O_2^{\cdot-}]_i}{dt} = \frac{V_{mito}}{V_{cyto}} J_{ROS}^{Tr} - J_{SOD,i}$$

$$(17)\quad \frac{d[\mathrm{H_2O_2}]_m}{dt} = 0.5J_{SOD,m} - J_{dif,\mathrm{H_2O_2}} - J_{GPX,m} - J_{TxPX,m}$$

$$(18)\quad \frac{d[\mathrm{H_2O_2}]_\mathrm{i}}{dt} = 0.5J_{SOD,i} + \frac{V_{mito}}{V_{cyto}} J_{dif,\mathrm{H_2O_2}} - J_{GPX,i} - J_{TxPX,i} - J_{CAT}$$

$$(19)\quad \frac{d[\mathrm{GSH}]_m}{dt} = J_{GR,m} - J_{GPX,m} - J_{GRX,m} + J_{GST} - J_{PSSG,m}$$

$$(20)\quad \frac{d[\mathrm{GSH}]_\mathrm{i}}{dt} = J_{GR,i} - J_{GPX,i} - J_{GRX,i} + \frac{V_{mito}}{V_{cyto}} J_{GST} - J_{PSSG,i}$$

$$(21)\quad \frac{d[\mathrm{GSSG}]_m}{dt} = 0.5(J_{GPX,m} - J_{GR,m}) + J_{GRX,m}$$

$$(22)\quad \frac{d[\mathrm{TrxSH_2}]_m}{dt} = J_{TR,m} - J_{TPX,m}$$

$$(23)\quad \frac{d[\mathrm{TrxSH_2}]_\mathrm{i}}{dt} = J_{TR,i} - J_{TPX,i}$$

$$(24)\quad \frac{d[\mathrm{PSSG}]_m}{dt} = J_{PSSG,m} - J_{GRX,m}$$

$$(25)\quad \frac{d[\mathrm{PSSG}]_\mathrm{i}}{dt} = J_{PSSG,i} - J_{GRX,i}$$

High energy phosphates:

$$(26)\quad \frac{d[\mathrm{ADP}]_m}{dt} = J_{ANT} - J_{F1Fo} - J_{SL}$$

$$(27)\quad \frac{d[ATP]_{mito}}{dt} = -J_{ANT} + J_{F1Fo} + J_{SL}$$

$$(28)\quad ([\mathrm{ATP}]_m = \Sigma[\mathrm{A}]_m - [\mathrm{ADP}]_m)$$

$$(29)\quad V_{ims}\frac{d[\mathrm{ADP}]_\mathrm{ims}}{dt} = -J_{ANT} + J_{CK}^{Mi} + J_{diff}^{ATP}$$

$$(30)\quad V_{ims}\frac{d[ATP]_{ims}}{dt} = J_{ANT} - J_{CK}^{Mi} - J_{diff}^{ATP}$$

$$(31)\quad ([\mathrm{ATP}]_\mathrm{ims} = \Sigma[\mathrm{A}]_\mathrm{ims} - [\mathrm{ADP}]_\mathrm{ims})$$

$$(32)\quad V_{cyto}\frac{d[ADP]_{cyt}}{dt} = J_{hyd} + J_{CK}^{MM} - J_{diff}^{ATP}$$

$$(33)\quad V_{cyto}\frac{d[ATP]_{cyt}}{dt} = -J_{hyd} - J_{CK}^{MM} + J_{diff}^{ATP}$$

$$(34)\quad ([\mathrm{ATP}]_\mathrm{cyt} = \Sigma[\mathrm{A}]_\mathrm{cyt} - [\mathrm{ADP}]_\mathrm{cyt})$$

$$(35)\quad V_{ims}\frac{d[\mathrm{Cr}]_\mathrm{ims}}{dt} = -J_{CK}^{Mi} + J_{diff}^{PCr}$$

$$(36)\quad V_{ims}\frac{d[\mathrm{PCr}]_{\mathrm{ims}}}{dt} = J_{CK}^{Mi} - J_{diff}^{PCr}$$
$$(37)\quad ([\mathrm{PCr}]_{\mathrm{ims}} = \Sigma[\mathrm{Cr}]_{\mathrm{ims}} - [\mathrm{Cr}]_{\mathrm{ims}})$$
$$(38)\quad V_{cyt}\frac{d[\mathrm{Cr}]_{\mathrm{cyt}}}{dt} = -J_{CK}^{MM} - J_{diff}^{PCr}$$
$$(39)\quad V_{cyt}\frac{d[\mathrm{PCr}]_{\mathrm{cyt}}}{dt} = J_{CK}^{MM} + J_{diff}^{PCr}$$
$$(40)\quad ([\mathrm{PCr}]_{\mathrm{cyt}} = \Sigma[\mathrm{Cr}]_{\mathrm{cyt}} - [\mathrm{Cr}]_{\mathrm{cyt}})$$
$$(41)\quad V_{ims}\frac{d[\mathrm{Pi}]_{\mathrm{ims}}}{dt} = -J_{PiC} - J_{diff}^{Pi}$$
$$(42)\quad V_{cyt}\frac{d[\mathrm{Pi}]_{\mathrm{cyt}}}{dt} = J_{hyd} + J_{diff}^{Pi}$$

Q cycle:

$$(43)\quad \frac{d[Q]_n}{dt} = v_5 - v_{7,ox} - v_{7,rd} - v_1$$
$$(44)\quad \frac{d[Q^{\cdot -}]_n}{dt} = v_{7,ox} + v_{7,rd} - v_{8,ox} - v_{8,rd}$$
$$(45)\quad \frac{d[QH_2]_n}{dt} = v_{8,ox} + v_{8,rd} + v_1 - v_2$$
$$(46)\quad \frac{d[QH_2]_p}{dt} = v_2 - v_3$$
$$(47)\quad \frac{d[Q^{\cdot -}]_p}{dt} = v_3 - v_{10} - v_{10b} - v_{4,ox} - v_{4,rd}$$
$$(48)\quad \frac{d[Q]_p}{dt} = v_{10} + v_{10b} + v_{4,ox} + v_{4,rd} - v_5$$
$$(49)\quad \frac{d[b1]}{dt} = v_{7,ox} + v_{8,ox} - v_{4,ox}$$
$$(50)\quad \frac{d[b2]}{dt} = v_{4,ox} + v_{7,rd} - v_{8,rd} - v_6$$
$$(51)\quad \frac{d[b3]}{dt} = v_6 - v_{4,rd} + v_{7,ox} - v_{8,ox}$$
$$(52)\quad (\frac{d[b4]}{dt} = v_{4,rd} - v_{7,rd} - v_{8,rd})$$
$$(53)\quad \frac{d[FeS]_{ox}}{dt} = v_9 - v_3$$
$$(54)\quad \frac{d[cytc1]_{ox}}{dt} = v_{33} - v_9$$
$$(55)\quad \frac{d[cytc]_{ox}}{dt} = V_e - v_{33}$$

The ATP hydrolysis pulse [2]:

$$(56)\quad J_{hyd} = H_{ATP,max}\ 6t/t_{cycle} \quad for \quad 0 < t < \frac{t_{cycle}}{6}$$
$$(57)\quad J_{hyd} = H_{ATP,max}\ [1 - 6(t/t_{cycle} - 1/6)] \quad for \quad \frac{t_{cycle}}{6} < t < \frac{t_{cycle}}{3}$$
$$(58)\quad J_{hyd} = 0 \quad for \quad \frac{t_{cycle}}{3} < t < t_{cycle}$$
$$(59)\quad H_{ATP,max} = 0.004mM/ms$$
$$(60)\quad t_{cycle} = 120ms$$

Cytosolic Ca^{2+} pulse:

(61) $Ca_i = t/t_{cycle}(Ca_{i,Amp} - Ca_{i,rest})/t_{Ca,peak} + Ca_{i,rest} \quad for \quad 0 < t < t_{Ca,peak}$

(62) $Ca_i = Ca_{i,Amp} - (Ca_{i,Amp} - Ca_{i,rest})(t/t_{cycle} - t_{Ca,peak})/(t_{cycle} - t_{Ca,peak}) \quad for \ t > t_{Ca,peak}$

(63) $Ca_{i,Amp} = 0.0004mM$

(64) $Ca_{i,rest} = 0.0001mM^*$

(65) $t_{cycle} = 120ms$

(66) $t_{Ca,peak} = 0.3t_{cycle}$

* The cytosolic calcium concentration is based on the Indo-1 calcium transient measurements obtained from isolated mouse cardiac myocytes[8].

The initial conditions used for calculations are as follows:

State variable	Value	Unit
$[Ca^{2+}]_m$	0.0677	µM
$[ADP]_m$	87.268	µM
$\Delta\Psi_m$	165.88	mV
$[NADH]$	956.7	µM
$[H^+]_m$	0.0292	µM
$[Pi]_m$	620.21	µM
$[ISOC]$	115.85	µM
$[\alpha KG]$	130.01	µM
$[SCoA]$	52.36	µM
$[SUC]$	22.46	µM
$[FUM]$	159.46	µM
$[MAL]$	149.74	µM
$[OAA]$	3.892	µM
$[NADPH]_m$	99.08	µM
$[O_2{}^-]_m$	9.23E-3	µM
$[O_2{}^-]_i$	2.93E-5	µM
$[H_2O_2]_m$	0.396	µM
$[H_2O_2]_i$	3.76E-4	µM

$[\mathrm{GSH}]_m$	3.82	µM
$[\mathrm{GSH}]_\mathrm{i}$	16.70	µM
$[\mathrm{TrxSH_2}]_m$	13.71	µM
$[\mathrm{TrxSH_2}]_i$	49.70	µM
$[\mathrm{GSSG}]_m$	2986.88	µM
$[\mathrm{PSSG}]_m$	0.9999	µM
$[\mathrm{PSSG}]_\mathrm{i}$	0.4509	µM
$[Q]_n$	1909	µM
$[Q^{\cdot -}]_n$	110.7	µM
$[QH_2]_n$	24.49	µM
$[QH_2]_p$	24.46	µM
$[Q^{\cdot -}]_p$	21.77	µM
$[Q]_p$	1909	µM
$[b1]$	226.89	µM
$[b2]$	68.83	µM
$[b3]$	28.26	µM
$[FeS]_{ox}$	192.95	µM
$[cytc1]_{ox}$	277.60	µM
$[cytc]_{ox}$	240.19	µM
$[ATP]_m$	1.046	mM
$[ADP]_{ims}$	0.0761	mM
$[ATP]_{ims}$	3.368	mM
$[Cr]_{ims}$	5.544	mM
$[PCr]_{ims}$	8.074	mM
$[ADP]_{cyt}$	0.0682	mM
$[ATP]_{cyt}$	3.376	mM

$[Cr]_{cyt}$	5.549	mM
$[PCr]_{cyt}$	8.069	mM
$[Pi]_{ims}$	1.598	mM
$[Pi]_{cyt}$	1.608	mM

General parameters used are as follows:

Parameter	Value	Unit	Description
F	96485	C/mol	Faraday constant
T	310	K	Absolute temperature
R	8.314	J/molK	Universal gas constant
V_T	26.71	mV	Thermal voltage RT/F)
C_m	1.0	$\mu\text{F}/\text{cm}^2$	Plasma membrane capacitance
C_{mito}	1.812	mM/V	Mitochondrial inner membrane capacitance
δ_{Ca}	0.0003	-	Mitochondrial free calcium fraction
δ_H	1E-5	-	Mitochondrial proton buffering factor

Fixed concentrations used are as follows:

Parameter	Value	Unit	Description
pH_i	7		Cytosolic pH
pH_m	7.3-7.8		Mitochondrial pH
$[\text{O}_2]$	0.006	mM	Tissue oxygen concentration
$[\text{Mg}^{2+}]_i$	1.0	mM	Cytosolic magnesium concentration
$[\text{Mg}^{2+}]_m$	0.4	mM	Mitochondrial magnesium concentration
$[\text{Na}^+]_i$	10	mM	Cytosolic sodium concentration
$\Sigma[\text{Pi}]_m$	8.6512	mM	Sum of mitochondrial inorganic phosphate

$\Sigma[\mathrm{N}]$	1	mM	Sum of mitochondrial NAD and NADH
$\Sigma[\mathrm{NADP}]_m$	0.1	mM	Sum of mitochondrial NADPH plus NADP
$[\mathrm{Ca}^{2+}]_\mathrm{i}$	1E-4 -4E-4	mM	Cytosolic calcium concentration

The creatine kinase models for both mitochondrial IMS and cytosolic compartments are based on a sequential, rapid equilibrium, random bi-bi enzymatic reaction scheme [2].

$$(67)\quad \mathrm{ATP} + \mathrm{Cr} \rightleftharpoons \mathrm{ADP} + \mathrm{PCr}$$

$$
\begin{aligned}
(68)\quad J_{CK} &= C_{CK}(F_{CK}V_f AB' - V_b PQ)/\Delta \\
(69)\quad \Delta &= 1 + A + B + P + Q + AB' + P'Q + PB'' \\
(70)\quad A &= [\mathrm{ATP}]/K_{ia} \\
(71)\quad B &= [\mathrm{Cr}]/K_{ib} \\
(72)\quad B' &= [\mathrm{Cr}]/K_b \\
(73)\quad B'' &= [\mathrm{Cr}]/K_{Ib} \\
(74)\quad P &= [\mathrm{ADP}]/K_{ic} \\
(75)\quad P' &= [\mathrm{ADP}]/K_c \\
(76)\quad Q &= [\mathrm{PCr}]/K_{id}
\end{aligned}
$$

Mitochondrial CK (CKmito) [6]:

Parameter	Value	Unit
V_f	0.775	mM * Hz
V_b	$K_f K_{CK,eq} K_{ic} K_d / K_{ia} K_b$	mM * Hz
K_{ia}	0.75132	mM
K_b	5.20908	mM
K_{ic}	0.2048	mM
K_d	0.49951	mM
K_{ib}	28.73344	mM
K_{id}	1.59769	mM
K_c	$K_{ic} K_d / K_{id}$	mM
K_{Ib}	K_{ib}	mM
$K_{CK,eq}$	151.95	
C_{CK}	0.25	(adjusted)

F_{CK}	25	(adjusted)

Cytosolic CK (CK-M) [6]:

Parameter	Value	Unit
V_f	7.37307	mM * Hz
V_b	$K_f K_{CK,eq} K_{ic} K_d / K_{ia} K_b$	mM * Hz
K_{ia}	1.02624	mM
K_b	16.7444	mM
K_{ic}	0.21226	mM
K_d	1.66976	mM
K_{ib}	34.50419	mM
K_{id}	4.51655	mM
K_c	$K_{ic} K_d / K_{id}$	mM
K_{Ib}	K_{ib}	mM
$K_{CK,eq}$	151.95	
C_{CK}	3	(adjusted)
F_{CK}	12	(adjusted)

Creatine shuttle [6]:

Parameter	Value	Unit	Description
R_{ATP}	8.16	Hz	conductance of ATP
R_{ADP}	R_{ATP}	Hz	conductance of ADP
R_{PCr}	14.6	Hz	conductance of PCr
R_{Cr}	R_{PCr}	Hz	conductance of Cr
R_{Pi}	18.4	Hz	conductance Pi

V_{cyto}	3	-	Relative volume of cytosol.<br>Volume of 1 correspondsto V_{mito}=0.153mL/gww.
V_{mito}	1	-	Relative volume of mitochondrial matrix.
V_{ims}	0.25	-	Relative volume of intermembrane space.

TCA cycle rates were calculated as follows [9]:

Citrate synthase (CS):

$$(77) \quad J_{CS} = \frac{k_{cat} E_T A B}{(1+A)(1+B)}$$

$$(78) \quad A = [\mathrm{AcCoA}]/K_m^{AcCoA}$$

$$(79) \quad B = [\mathrm{OAA}]/K_m^{OAA}$$

Parameter	Value	Unit	Description
k_{cat}	0.23523	Hz	Catalytic constant
E_T	400	μM	Enzyme concentration of CS
K_m^{AcCoA}	12.6	μM	Michaelis constant for AcCoA
K_m^{OAA}	0.64	μM	Michaelis constant for OAA
$[\mathrm{AcCoA}]$	1000	μM	Acetyl CoA concentration
k_{cat} (cell)	0.15891	Hz	Catalytic constant (cellular model)

Aconitase (ACO):

$$(80) \quad J_{ACO} = k_f([CIT] - [ISOC]/K_{eq})$$

$$(81) \quad [CIT] = \Sigma_{CAC} - [ISOC] - [\alpha KG] - [SCoA] - [SUC] - [FUM] - [MAL] - [OAA]$$

Parameter	Value	Unit	Description
k_f	0.11688	Hz	Forward rate constant of ACO
K_{eq}	2.22	-	Equilibrium constant of ACO
Σ_{CAC}	1300	μM	Sum of TCA cycle intermediates
k_f (cell)	0.078959	Hz	Forward rate constant (cellular model)

Isocitrate dehydrogenase, NADH-producing (IDH3):

$$(82)\quad J_{IDH3} = \frac{k_{cat}E_T AB}{f_H AB + f_i B + f_a A + f_a f_i}$$

$$(83)\quad f_H = 1 + \frac{[H^+]_m}{K_{H1}} + \frac{K_{H2}}{[H^+]_m}$$

$$(84)\quad A = [NAD]/K_{NAD}$$

$$(85)\quad B = ([ISOC]/K_{ISOC})^n$$

$$(86)\quad f_a = \frac{K_A}{K_A + [ADP]_m}\frac{K_{CA}}{K_{CA} + [Ca^{2+}]_m}$$

$$(87)\quad f_i = 1 + \frac{[NADH]}{K_{NADH}}$$

Parameter	Value	Unit	Description
k_{cat}	11.88	kHz	Rate constant of IDH3
E_T	109	µM	Concentration of IDH3
K_{H1}	1E-9	M	Ionization constant of IDH3
K_{H2}	9E-7	M	Ionization constant of IDH3
K_{NAD}	923	µM	Michaelis constant for NAD
K_{ISOC}	1520	µM	Michaelis constant for isocitrate
n	2	-	Cooperativity for isocitrate
K_A	620	µM	Activation constant by ADP
K_{CA}	0.5	µM	Activation constant for calcium
K_{NADH}	190	µM	Inhibition constant by NADH
k_{cat} (cell)	535	Hz	Rate constant (cellular model)

Alpha-ketoglutarate dehydrogenase (KGDH)

$$(88)\quad J_{KGDH} = \frac{k_{cat}E_T AB}{f_H AB + f_a(A + B)}$$

$$(89)\quad f_H = 1 + \frac{[H^+]_m}{K_{H1}} + \frac{K_{H2}}{[H^+]_m}$$

$$(90)\quad A = [NAD]/K_{NAD}$$

$$(91)\quad B = ([\alpha KG]/K_{AKG})^n$$

$$(92)\quad f_a = \frac{K_{MG}}{K_{MG} + [Mg^{2+}]_m}\frac{K_{CA}}{K_{CA} + [Ca^{2+}]_m}$$

Parameter	Value	Unit	Description
k_{cat}	13.2	Hz	Rate constant of KGDH
E_T	500	µM	Concentration of KGDH
K_{H1}	4E-8	M	Ionization constant of KGDH
K_{H2}	7E-8	M	Ionization constant of KGDH
K_{NAD}	38700	µM	Michaelis constant for NAD
K_{AKG}	30000	µM	Michaelis constant for αKG
n	1.2	-	Hill coefficient for αKG
K_{MG}	30.8	µM	Activation constant for Mg
K_{CA}	0.15	µM	Activation constant for Ca
(cell)	17.9	Hz	Rate constant (cellular model)

Succinate-CoA ligase (SL)

$$(93) \quad J_{SL} = k_f([SCoA][ADP]_m[Pi]_m - [SUC][ATP]_m[CoA]/K_{eq}^{app})$$

$$(94) \quad K_{eq}^{app} = K_{eq}\frac{P_{SUC}P_{ATP}}{P_{Pi}P_{ADP}}$$

Parameter	Value	Unit	Description
k_f	0.028	µM * Hz	Forward rate constant of SL
K_{eq}	3.115	-	Equilibrium constant of SL
[CoA]	20	µM	Coenzyme A concentration
k_f (cell)	0.0284	µM * Hz	Forward rate constant (cellular model)

Succinate dehydrogenase (SDH)

See electron transport chain.

Fumarate hydratase (FH)

$$(95) \quad J_{FH} = k_f([FUM] - [MAL]/K_{eq})$$

Parameter	Value	Unit	Description
k_f	8.3	Hz	Forward rate constant
K_{eq}	1.0	-	Equilibrium constant
k_f (cell)	8.4	Hz	Forward rate constant (cellular model)

Malate dehydrogenase(MDH)

$$(96)\quad J_{MDH} = \frac{k_{cat} E_T A B f_a f_i}{(1+A)(1+B)}$$

$$(97)\quad A = \frac{[MAL]}{K_{MAL}} \frac{K_{OAA}}{K_{OAA} + [OAA]}$$

$$(98)\quad B = [NAD]/K_{NAD}$$

$$(99)\quad f_a = k_{offset} + \left(1 + \frac{[H^+]_m}{K_{H1}}(1 + \frac{[H^+]_m}{K_{H2}})\right)^{-1}$$

$$(100)\quad f_i = \left(1 + \frac{K_{H3}}{[H^+]_m}(1 + \frac{K_{H4}}{[H^+]_m})\right)^2$$

Parameter	Value	Units	Description
k_{cat}	124.2	Hz	Rate constant
E_T	154	µM	
K_{H1}	1.131E-8	M	Ionization constant
K_{H2}	2.67E-2	M	Ionization constant
K_{H3}	6.68E-12	M	Ionization constant
K_{H4}	5.62E-9	M	Ionization constant
k_{offset}	0.0399		Offset of MDH pH activation factor
K_{NAD}	224.4	µM	Michaelis constant for NAD
K_{MAL}	1493	µM	Michaelis constant for malate
K_{OAA}	31	µM	Inhibition constant for oxaloacetate
k_{cat} (cell)	125.9	Hz	Rate constant for cellular model

Aspartate aminotransferase(AAT)

$$(101)\ J_{AAT} = k_f[OAA][GLU]\frac{k_{ASP}K_{eq}}{k_{ASP}K_{eq}+k_f[\alpha KG]}$$

Parameter	Value	Units	Description
k_f	21.4	Hz	Forward rate constant
k_{ASP}	0.0015	Hz	Rate constant of aspartate consumption
K_{eq}	6.6		Equilibrium constant
[GLU]	30000	µM	Glutamate concentration
k_f (cell)	21.7	Hz	Forward rate constant (cellular model)

Acid-base equilibria and binding polynomials for both cytoplasmic and mitochondrial compartments were calculated as follows [9]:

$$(102)\quad P_{ATP} = 1+\frac{[\mathrm{H}^+]}{K_a^{ATP}}+\frac{[\mathrm{Mg}^{2+}]}{K_{Mg}^{ATP}}$$

$$(103)\quad P_{ADP} = 1+\frac{[\mathrm{H}^+]}{K_a^{ADP}}+\frac{[\mathrm{Mg}^{2+}]}{K_{Mg}^{ADP}}$$

$$(104)\quad P_{Pi} = 1+\frac{[\mathrm{H}^+]}{K_a^{Pi}}$$

$$(105)\quad P_{SUC} = 1+\frac{[\mathrm{H}^+]}{K_a^{SUC}}$$

$$(106)\quad P_{H_2O} = 1+\frac{[\mathrm{H}^+]}{K_w}$$

$$(107)\quad [\mathrm{OH}^-] = K_A^{H_2O}/[\mathrm{H}^+]$$

$$(108)\quad [\mathrm{ATP}^{4-}] = \Sigma ATP/P_{ATP}$$

$$(109)\quad [\mathrm{ADP}^{3-}] = \Sigma ADP/P_{ADP}$$

$$(110)\quad [\mathrm{HPO_4}^{2-}] = \Sigma Pi/P_{Pi}$$

$$(111)\quad [\mathrm{HATP}^{3-}] = [\mathrm{ATP}^{4-}]\frac{[\mathrm{H}^+]}{K_a^{ATP}}$$

$$(112)\quad [\mathrm{HADP}^{2-}] = [\mathrm{ADP}^{3-}]\frac{[\mathrm{H}^+]}{K_a^{ADP}}$$

$$(113)\quad [\mathrm{H_2PO_4}^{-}] = [\mathrm{HPO_4}^{2-}]\frac{[\mathrm{H}^+]}{K_a^{Pi}}$$

$$(114)\quad [\mathrm{MgATP}^{2-}] = [\mathrm{ATP}^{4-}]\frac{[\mathrm{Mg}^{2+}]}{K_{Mg}^{ATP}}$$

$$(115)\quad [\mathrm{MgADP}^{-}] = [\mathrm{ADP}^{3-}]\frac{[\mathrm{Mg}^{2+}]}{K_{Mg}^{ADP}}$$

Parameter	Value	Unit	Description
δ_H	1E-5	-	mitochondria buffering capacity
pK_a^{ATP}	6.48	-	pK of ATP acid dissociation constant
pK_a^{ADP}	6.38	-	pK of ADP acid dissociation constant
pK_a^{Pi}	6.75	-	pKa of phosphate acid dissociation constant
pK_{Mg}^{ATP}	4.19	-	pK of ATP magnesium dissociation constant
pK_{Mg}^{ADP}	3.25	-	pK of ADP magnesium dissociation constant
pK_a^{SUC}	5.2	-	pK of succinic acid dissociation constant
pKw	14		pK of water acid dissociation constant

Mitochondrial transporters and ion channels we calculated as follows:

Phosphate carrier [9] follows equilibrium random Bi:Bi reaction kinetics.

$$(116)\quad J_{PiC} = c_{PiC}(V_f AB - V_b PQ)/\Delta$$
$$(117)\quad A = [\mathrm{HPO_4}^{2-}]_i/K_{pi,i}$$
$$(118)\quad P = [\mathrm{HPO_4}^{2-}]_m/K_{pi,m}$$
$$(119)\quad Q = [\mathrm{OH}^-]_i/K_{OH,i}$$
$$(120)\quad B = [\mathrm{OH}^-]_m/K_{OH,m}$$
$$(121)\quad \Delta = 1 + A + B + P + Q + AB + PQ$$
$$(122)\quad V_b = \frac{V_f K_{pi,m} K_{OH,i}}{K_{eq} K_{pi,i} K_{OH,m}}$$

Parameter	Value	Unit	Description
$K_{pi,i}$	11.06	mM	Extra-matrix Pi binding constant
$K_{pi,m}$	11.06	mM	Mitochondrial matrix Pi binding constant
$K_{OH,i}$	4.08E-5	mM	Extra-matrix OH- binding constant
$K_{OH,m}$	4.08E-5	mM	Mitochondrial matrix OH- binding constant
V_f	1.5	Hz	Forward rate
c_{PiC}	4.9	mM	PiC activity
K_{eq}	1	-	Equilibrium constant of PiC

Mitochondrial Sodium-hydrogen exchanger [9] follows Smith and Crampin's model of counterpart on the plasma membrane.

$$(123)\quad J_{NHE} = c_{NHE} f_h \frac{\beta_1^+ \beta_2^+ - \beta_1^- \beta_2^-}{\beta_1^+ + \beta_2^+ + \beta_1^- + \beta_2^-}$$

$$(124)\quad f_h = \frac{([\mathrm{H}^+]_i)^n}{([\mathrm{H}^+]_i)^n + K_i^n}$$

$$(125)\quad A = \frac{[\mathrm{Na}^+]_m}{K_{Na}}$$

$$(126)\quad B = \frac{[\mathrm{H}^+]_i}{K_H}$$

$$(127)\quad P = \frac{[\mathrm{Na}^+]_i}{K_{Na}}$$

$$(128)\quad Q = \frac{[\mathrm{H}^+]_m}{K_H}$$

$$(129)\quad \beta_1^+ = \frac{k_1^+ A}{1 + A + Q}$$

$$(130)\quad \beta_1^- = \frac{k_1^- P}{1 + P + B}$$

$$(131)\quad \beta_2^+ = \frac{k_4^+ B}{1 + P + B}$$

$$(132)\quad \beta_2^- = \frac{k_4^- Q}{1 + A + Q}$$

$$(133)\quad k_4^- = \frac{k_1^+ k_4^+}{K_{eq} k_1^-}$$

Parameter	Value	Unit	Description
c_{NHE}	0.00785	mM	NHE concentration
K_{Na}	24	mM	Na Dissociation constant
K_H	158.5	nM	H Dissociation constant
K_i	3.02	nM	Proton binding constant
n	3	-	Hill coefficient for H+ binding
k_1^+	25.2	Hz	NHE forward rate constant
k_1^-	42.9	Hz	NHE backward rate constant
k_4^+	160	Hz	NHE forward rate constant
K_{eq}	1	-	Equilibrium constant of NHE

Adenine Nucleotide translocator (ANT) [9]

$$(134) \quad J_{ANT} = V_{max} \frac{AB - \delta PQ}{(B + \delta^h P)(A + Q)}$$
$$(135) \quad A = [\mathrm{ATP}^{4-}]_m$$
$$(136) \quad B = [\mathrm{ADP}^{3-}]_i$$
$$(137) \quad P = [\mathrm{ATP}^{4-}]_i$$
$$(138) \quad Q = [\mathrm{ADP}^{3-}]_m$$
$$(139) \quad \delta = \exp(-F\Delta\Psi_m/V_T)$$

Parameter	Value	Unit	Description
V_{max}	3150	mM * Hz	Maximal rate
h	0.5	-	Fraction of dpsi

Mitochondrial calcium uniporter (MCU)[9]

$$(140) \quad J_{uni} = V_{max} \frac{S(1+S)^3}{(1+S)^4 + L(1+A)^n} \frac{\delta}{e^{\delta} - 1}$$
$$(141) \quad S = [\mathrm{Ca}^{2+}]_i/K_{trans}$$
$$(142) \quad A = [\mathrm{Ca}^{2+}]_i/K_{act}$$
$$(143) \quad \delta = -Z_{Ca}(\Delta\Psi_m - \Delta\Psi_0)/V_T$$

Parameter	Value	Unit	Description
V_{max}	1.5	mM*Hz	Maximal rate (adjusted)
$\Delta\Psi_0$	91	mV	Offset potential
K_{act}	3.8E-4	mM	Activation constant for calcium
K_{trans}	0.019	mM	Dissociation constant for calcium
n	-2.8	-	Activation cooperativity
L	110	-	Keq for conformational transitions

Mitochondrial sodium-calcium exchanger (NCLX) [9]

$$(144) \quad J_{NCLX} = V_{max} \exp(b\Delta\Psi_m/V_T) \frac{[Ca^{2+}]_m}{[Ca^{2+}]_i} (\frac{A}{1+A})^n \frac{B}{1+B}$$
$$(145) \quad A = [\mathrm{Na}^{+}]_i/K_{Na}$$
$$(146) \quad B = [\mathrm{Ca}^{2+}]_m/K_{Ca}$$

Parameter	Value	Unit	Description
V_{max}	0.183	mM* Hz	
b	0.5	-	
K_{Na}	9.4	mM	
K_{Ca}	0.375	µM	
n	3		

Mitochondrial proton leak

$$(147)\ J_{hleak} = g_H \Delta\Psi_m$$

Parameter	Value	Unit	Description
g_H	2	mM*Hz/V	Ionic conductance of the inner membrane

Mitochondrial hydrogen flux balance [9]

J_H : Proton influx to mitochondrial matrix by pumps / transporters

J_{Hn} : Proton flux due to enzyme stoichiometry

J_{HL} : Proton flux due to ligand binding / unbinding

$$(148)\quad J_H = -J_{He} - J_{HSDH} + J_{hu} + J_{NHE} + J_{PiC} + J_{Hleak}$$

$$(149)\quad J_{Hn} = -(J_{IDH} + J_{KGDH} + J_{MDH} - J_{F1Fo})$$

$$(150)\quad J_{HL} = \frac{[H^+]_m}{K_{a,ATP}P_{ATP}}\frac{d[ATP]_m}{dt} + \frac{[H^+]_m}{K_{a,ADP}P_{ADP}}\frac{d[ADP]_m}{dt} + \frac{[H^+]_m}{K_{a,Pi}P_{Pi}}\frac{d[Pi]_m}{dt} + \frac{[H^+]_m}{K_{a,SUC}P_{SUC}}\frac{d[SUC]_m}{dt}$$

$$(151)\quad \frac{d[H^+]_m}{dt} = \delta_H(J_H - J_{Hn} - J_{HL})$$

Mitochondrial electron transport was calculated as follows:

Complex I [3], assuming single electron for each cycle.

$$(152)\quad \nu = \exp((\Delta\Psi_m - \Delta\Psi_B)/V_T)$$

$$(153)\quad a_{12} = k_{12}([H^+]_m)^2$$

$$(154)\quad a_{21} = k_{21}$$

$$(155)\quad a_{65} = k_{65}([H^+]_i)^2$$

$$(156)\quad a_{56} = k_{56}$$

$$(157)\quad a_{61} = k_{61}/\nu$$

$$(158)\quad a_{16} = k_{16}\nu$$

$$a_{23} = k_{23}\sqrt{[NADH]} \tag{159}$$
$$a_{32} = k_{32} \tag{160}$$
$$a_{34} = k_{34} \tag{161}$$
$$a_{43} = k_{43}\sqrt{[NAD^+]} \tag{162}$$
$$a_{47} = C1_{inhib} \cdot K_{47}\sqrt{[Q_n][H^+]_m} \tag{163}$$
$$a_{74} = k_{74} \tag{164}$$
$$a_{57} = C1_{inhib} \cdot K_{57}\sqrt{[QH_2]} \tag{165}$$
$$a_{75} = k_{75} \tag{166}$$
$$k'_{42} = k_{42}(1 + k_{RC}[DOX]) \tag{167}$$
$$a_{42} = k'_{42}[O_2] \tag{168}$$
$$K_{eq}^{ROS} = \exp((E_{FMN} - E_{sox})/V_T) \tag{169}$$
$$a_{24} = a_{42}K_{eq}^{ROS}[O_2^{\cdot -}]_m \tag{170}$$
$$a_{25} = a_{52} = 0 \tag{171}$$
$$\Delta = e_1 + e_2 + e_3 + e_4 + e_5 + e_6 + e_7 \tag{172}$$
$$\rho'_{C1} = \rho_{C1} \cdot mt_{prot}/\Delta \tag{173}$$
$$J_{Hres}^{C1} = 2\rho'_{C1}(e_6 a_{61} - e_1 a_{16}) \tag{174}$$
$$J_Q^{C1} = 0.5\rho'_{C1}(e_4 a_{47} - e_7 a_{74}) \tag{175}$$
$$J_{NADH}^{C1} = 0.5\rho'_{C1}(e_3 a_{34} - e_4 a_{43}) \tag{176}$$
$$J_{ROS}^{C1} = \rho'_{C1}(e_4 a_{42} - e_2 a_{24}) \tag{177}$$

$$\begin{aligned} e_1 = {} & a_{21} \cdot a_{32} \cdot a_{42} \cdot a_{56} \cdot a_{61} \cdot a_{74} + a_{21} \cdot a_{32} \cdot a_{42} \cdot a_{56} \cdot a_{61} \cdot a_{75} + a_{21} \cdot a_{32} \cdot a_{42} \cdot a_{57} \cdot a_{61} \cdot a_{74} \\ & + a_{21} \cdot a_{32} \cdot a_{42} \cdot a_{57} \cdot a_{65} \cdot a_{74} + a_{21} \cdot a_{32} \cdot a_{43} \cdot a_{56} \cdot a_{61} \cdot a_{74} + a_{21} \cdot a_{32} \cdot a_{43} \cdot a_{56} \cdot a_{61} \cdot a_{75} \\ & + a_{21} \cdot a_{32} \cdot a_{43} \cdot a_{57} \cdot a_{61} \cdot a_{74} + a_{21} \cdot a_{32} \cdot a_{43} \cdot a_{57} \cdot a_{65} \cdot a_{74} + a_{21} \cdot a_{32} \cdot a_{47} \cdot a_{56} \cdot a_{61} \cdot a_{75} \\ & + a_{21} \cdot a_{34} \cdot a_{42} \cdot a_{56} \cdot a_{61} \cdot a_{74} + a_{21} \cdot a_{34} \cdot a_{42} \cdot a_{56} \cdot a_{61} \cdot a_{75} + a_{21} \cdot a_{34} \cdot a_{42} \cdot a_{57} \cdot a_{61} \cdot a_{74} \\ & + a_{21} \cdot a_{34} \cdot a_{42} \cdot a_{57} \cdot a_{65} \cdot a_{74} + a_{21} \cdot a_{34} \cdot a_{47} \cdot a_{56} \cdot a_{61} \cdot a_{75} + a_{23} \cdot a_{34} \cdot a_{47} \cdot a_{56} \cdot a_{61} \cdot a_{75} \\ & + a_{24} \cdot a_{32} \cdot a_{47} \cdot a_{56} \cdot a_{61} \cdot a_{75} + a_{24} \cdot a_{34} \cdot a_{47} \cdot a_{56} \cdot a_{61} \cdot a_{75} \end{aligned} \tag{178}$$

$$\begin{aligned} e_2 = {} & a_{12} \cdot a_{32} \cdot a_{42} \cdot a_{56} \cdot a_{61} \cdot a_{74} + a_{12} \cdot a_{32} \cdot a_{42} \cdot a_{56} \cdot a_{61} \cdot a_{75} + a_{12} \cdot a_{32} \cdot a_{42} \cdot a_{57} \cdot a_{61} \cdot a_{74} \\ & + a_{12} \cdot a_{32} \cdot a_{42} \cdot a_{57} \cdot a_{65} \cdot a_{74} + a_{12} \cdot a_{32} \cdot a_{43} \cdot a_{56} \cdot a_{61} \cdot a_{74} + a_{12} \cdot a_{32} \cdot a_{43} \cdot a_{56} \cdot a_{61} \cdot a_{75} \\ & + a_{12} \cdot a_{32} \cdot a_{43} \cdot a_{57} \cdot a_{61} \cdot a_{74} + a_{12} \cdot a_{32} \cdot a_{43} \cdot a_{57} \cdot a_{65} \cdot a_{74} + a_{12} \cdot a_{32} \cdot a_{47} \cdot a_{56} \cdot a_{61} \cdot a_{75} \\ & + a_{12} \cdot a_{34} \cdot a_{42} \cdot a_{56} \cdot a_{61} \cdot a_{74} + a_{12} \cdot a_{34} \cdot a_{42} \cdot a_{56} \cdot a_{61} \cdot a_{75} + a_{12} \cdot a_{34} \cdot a_{42} \cdot a_{57} \cdot a_{61} \cdot a_{74} \\ & + a_{12} \cdot a_{34} \cdot a_{42} \cdot a_{57} \cdot a_{65} \cdot a_{74} + a_{12} \cdot a_{34} \cdot a_{47} \cdot a_{56} \cdot a_{61} \cdot a_{75} + a_{16} \cdot a_{32} \cdot a_{42} \cdot a_{57} \cdot a_{65} \cdot a_{74} \\ & + a_{16} \cdot a_{32} \cdot a_{43} \cdot a_{57} \cdot a_{65} \cdot a_{74} + a_{16} \cdot a_{34} \cdot a_{42} \cdot a_{57} \cdot a_{65} \cdot a_{74} \end{aligned} \tag{179}$$

$$\begin{aligned} e_3 = {} & a_{12} \cdot a_{23} \cdot a_{42} \cdot a_{56} \cdot a_{61} \cdot a_{74} + a_{12} \cdot a_{23} \cdot a_{42} \cdot a_{56} \cdot a_{61} \cdot a_{75} + a_{12} \cdot a_{23} \cdot a_{42} \cdot a_{57} \cdot a_{61} \cdot a_{74} \\ & + a_{12} \cdot a_{23} \cdot a_{42} \cdot a_{57} \cdot a_{65} \cdot a_{74} + a_{12} \cdot a_{23} \cdot a_{43} \cdot a_{56} \cdot a_{61} \cdot a_{74} + a_{12} \cdot a_{23} \cdot a_{43} \cdot a_{56} \cdot a_{61} \cdot a_{75} \\ & + a_{12} \cdot a_{23} \cdot a_{43} \cdot a_{57} \cdot a_{61} \cdot a_{74} + a_{12} \cdot a_{23} \cdot a_{43} \cdot a_{57} \cdot a_{65} \cdot a_{74} + a_{12} \cdot a_{23} \cdot a_{47} \cdot a_{56} \cdot a_{61} \cdot a_{75} \\ & + a_{12} \cdot a_{24} \cdot a_{43} \cdot a_{56} \cdot a_{61} \cdot a_{74} + a_{12} \cdot a_{24} \cdot a_{43} \cdot a_{56} \cdot a_{61} \cdot a_{75} + a_{12} \cdot a_{24} \cdot a_{43} \cdot a_{57} \cdot a_{61} \cdot a_{74} \\ & + a_{12} \cdot a_{24} \cdot a_{43} \cdot a_{57} \cdot a_{65} \cdot a_{74} + a_{16} \cdot a_{21} \cdot a_{43} \cdot a_{57} \cdot a_{65} \cdot a_{74} + a_{16} \cdot a_{23} \cdot a_{42} \cdot a_{57} \cdot a_{65} \cdot a_{74} \\ & + a_{16} \cdot a_{23} \cdot a_{43} \cdot a_{57} \cdot a_{65} \cdot a_{74} + a_{16} \cdot a_{24} \cdot a_{43} \cdot a_{57} \cdot a_{65} \cdot a_{74} \end{aligned} \tag{180}$$

$$\begin{aligned} e_4 = {} & a_{12} \cdot a_{23} \cdot a_{34} \cdot a_{56} \cdot a_{61} \cdot a_{74} + a_{12} \cdot a_{23} \cdot a_{34} \cdot a_{56} \cdot a_{61} \cdot a_{75} + a_{12} \cdot a_{23} \cdot a_{34} \cdot a_{57} \cdot a_{61} \cdot a_{74} \\ & + a_{12} \cdot a_{23} \cdot a_{34} \cdot a_{57} \cdot a_{65} \cdot a_{74} + a_{12} \cdot a_{24} \cdot a_{32} \cdot a_{56} \cdot a_{61} \cdot a_{74} + a_{12} \cdot a_{24} \cdot a_{32} \cdot a_{56} \cdot a_{61} \cdot a_{75} \\ & + a_{12} \cdot a_{24} \cdot a_{32} \cdot a_{57} \cdot a_{61} \cdot a_{74} + a_{12} \cdot a_{24} \cdot a_{32} \cdot a_{57} \cdot a_{65} \cdot a_{74} + a_{12} \cdot a_{24} \cdot a_{34} \cdot a_{56} \cdot a_{61} \cdot a_{74} \\ & + a_{12} \cdot a_{24} \cdot a_{34} \cdot a_{56} \cdot a_{61} \cdot a_{75} + a_{12} \cdot a_{24} \cdot a_{34} \cdot a_{57} \cdot a_{61} \cdot a_{74} + a_{12} \cdot a_{24} \cdot a_{34} \cdot a_{57} \cdot a_{65} \cdot a_{74} \\ & + a_{16} \cdot a_{21} \cdot a_{32} \cdot a_{57} \cdot a_{65} \cdot a_{74} + a_{16} \cdot a_{21} \cdot a_{34} \cdot a_{57} \cdot a_{65} \cdot a_{74} + a_{16} \cdot a_{23} \cdot a_{34} \cdot a_{57} \cdot a_{65} \cdot a_{74} \\ & + a_{16} \cdot a_{24} \cdot a_{32} \cdot a_{57} \cdot a_{65} \cdot a_{74} + a_{16} \cdot a_{24} \cdot a_{34} \cdot a_{57} \cdot a_{65} \cdot a_{74} \end{aligned} \tag{181}$$

(182) $e_5 = a_{12} \cdot a_{23} \cdot a_{34} \cdot a_{47} \cdot a_{61} \cdot a_{75} + a_{12} \cdot a_{23} \cdot a_{34} \cdot a_{47} \cdot a_{65} \cdot a_{75} + a_{12} \cdot a_{24} \cdot a_{32} \cdot a_{47} \cdot a_{61} \cdot a_{75} + a_{12} \cdot a_{24} \cdot a_{32} \cdot a_{47} \cdot a_{65} \cdot a_{75} + a_{12} \cdot a_{24} \cdot a_{34} \cdot a_{47} \cdot a_{61} \cdot a_{75} + a_{12} \cdot a_{24} \cdot a_{34} \cdot a_{47} \cdot a_{65} \cdot a_{75} + a_{16} \cdot a_{21} \cdot a_{32} \cdot a_{42} \cdot a_{65} \cdot a_{74} + a_{16} \cdot a_{21} \cdot a_{32} \cdot a_{42} \cdot a_{65} \cdot a_{75} + a_{16} \cdot a_{21} \cdot a_{32} \cdot a_{43} \cdot a_{65} \cdot a_{74} + a_{16} \cdot a_{21} \cdot a_{32} \cdot a_{43} \cdot a_{65} \cdot a_{75} + a_{16} \cdot a_{21} \cdot a_{32} \cdot a_{47} \cdot a_{65} \cdot a_{75} + a_{16} \cdot a_{21} \cdot a_{34} \cdot a_{42} \cdot a_{65} \cdot a_{74} + a_{16} \cdot a_{21} \cdot a_{34} \cdot a_{42} \cdot a_{65} \cdot a_{75} + a_{16} \cdot a_{21} \cdot a_{34} \cdot a_{47} \cdot a_{65} \cdot a_{75} + a_{16} \cdot a_{23} \cdot a_{34} \cdot a_{47} \cdot a_{65} \cdot a_{75} + a_{16} \cdot a_{24} \cdot a_{32} \cdot a_{47} \cdot a_{65} \cdot a_{75} + a_{16} \cdot a_{24} \cdot a_{34} \cdot a_{47} \cdot a_{65} \cdot a_{75}$

(183) $e_6 = a_{12} \cdot a_{23} \cdot a_{34} \cdot a_{47} \cdot a_{56} \cdot a_{75} + a_{12} \cdot a_{24} \cdot a_{32} \cdot a_{47} \cdot a_{56} \cdot a_{75} + a_{12} \cdot a_{24} \cdot a_{34} \cdot a_{47} \cdot a_{56} \cdot a_{75} + a_{16} \cdot a_{21} \cdot a_{32} \cdot a_{42} \cdot a_{56} \cdot a_{74} + a_{16} \cdot a_{21} \cdot a_{32} \cdot a_{42} \cdot a_{56} \cdot a_{75} + a_{16} \cdot a_{21} \cdot a_{32} \cdot a_{42} \cdot a_{57} \cdot a_{74} + a_{16} \cdot a_{21} \cdot a_{32} \cdot a_{43} \cdot a_{56} \cdot a_{74} + a_{16} \cdot a_{21} \cdot a_{32} \cdot a_{43} \cdot a_{56} \cdot a_{75} + a_{16} \cdot a_{21} \cdot a_{32} \cdot a_{43} \cdot a_{57} \cdot a_{74} + a_{16} \cdot a_{21} \cdot a_{32} \cdot a_{47} \cdot a_{56} \cdot a_{75} + a_{16} \cdot a_{21} \cdot a_{34} \cdot a_{42} \cdot a_{56} \cdot a_{74} + a_{16} \cdot a_{21} \cdot a_{34} \cdot a_{42} \cdot a_{56} \cdot a_{75} + a_{16} \cdot a_{21} \cdot a_{34} \cdot a_{42} \cdot a_{57} \cdot a_{74} + a_{16} \cdot a_{21} \cdot a_{34} \cdot a_{47} \cdot a_{56} \cdot a_{75} + a_{16} \cdot a_{23} \cdot a_{34} \cdot a_{47} \cdot a_{56} \cdot a_{75} + a_{16} \cdot a_{24} \cdot a_{32} \cdot a_{47} \cdot a_{56} \cdot a_{75} + a_{16} \cdot a_{24} \cdot a_{34} \cdot a_{47} \cdot a_{56} \cdot a_{75}$

(184) $e_7 = a_{12} \cdot a_{23} \cdot a_{34} \cdot a_{47} \cdot a_{56} \cdot a_{61} + a_{12} \cdot a_{23} \cdot a_{34} \cdot a_{47} \cdot a_{57} \cdot a_{61} + a_{12} \cdot a_{23} \cdot a_{34} \cdot a_{47} \cdot a_{57} \cdot a_{65} + a_{12} \cdot a_{24} \cdot a_{32} \cdot a_{47} \cdot a_{56} \cdot a_{61} + a_{12} \cdot a_{24} \cdot a_{32} \cdot a_{47} \cdot a_{57} \cdot a_{61} + a_{12} \cdot a_{24} \cdot a_{32} \cdot a_{47} \cdot a_{57} \cdot a_{65} + a_{12} \cdot a_{24} \cdot a_{34} \cdot a_{47} \cdot a_{56} \cdot a_{61} + a_{12} \cdot a_{24} \cdot a_{34} \cdot a_{47} \cdot a_{57} \cdot a_{61} + a_{12} \cdot a_{24} \cdot a_{34} \cdot a_{47} \cdot a_{57} \cdot a_{65} + a_{16} \cdot a_{21} \cdot a_{32} \cdot a_{42} \cdot a_{57} \cdot a_{65} + a_{16} \cdot a_{21} \cdot a_{32} \cdot a_{43} \cdot a_{57} \cdot a_{65} + a_{16} \cdot a_{21} \cdot a_{32} \cdot a_{47} \cdot a_{57} \cdot a_{65} + a_{16} \cdot a_{21} choco \cdot a_{34} \cdot a_{42} \cdot a_{57} \cdot a_{65} + a_{16} \cdot a_{21} \cdot a_{34} \cdot a_{47} \cdot a_{57} \cdot a_{65} + a_{16} \cdot a_{23} \cdot a_{34} \cdot a_{47} \cdot a_{57} \cdot a_{65} + a_{16} \cdot a_{24} \cdot a_{32} \cdot a_{47} \cdot a_{57} \cdot a_{65} + a_{16} \cdot a_{24} \cdot a_{34} \cdot a_{47} \cdot a_{57} \cdot a_{65}$

Parameter	Value	Units	Description
ρ_{C1}	8849	μ	Concentration of complex I (Adjustable)
$\Delta\Psi_B$	50	mV	Phase boundary potential
k_{12}	6.3396E11	Hz/mM^2	
k_{21}	5	Hz	
k_{56}	100	Hz	
k_{65}	2.5119E13	Hz/mM^2	
k_{61}	1E7	Hz	
k_{16}	130	Hz	
k_{23}	3886.7	Hz/mM^{1/2}	
k_{32}	9.1295E6	Hz	
k_{34}	639.1364	Hz	
k_{43}	3.2882	Hz/mM^{1/2}	
k_{47}	1.5962E7	Hz/mM	
k_{74}	65.2227	Hz	

k_{75}	24615	Hz	
k_{57}	1166.7	Hz/mM^{1/2}	
k_{42}	6.0318	Hz/mM	
E_{FMN}	-0.375	V	Midpoint potential of flavin mononucleotide
E_{sox}	-0.15	V	Midpoint potential of superoxide

Complex II (Succinate dehydrogenase) [3]

$$(185) \quad f_Q = \frac{[Q]_n}{[Q]_n + [QH_2]_n}$$
$$(186) \quad f_{OAA} = \frac{K_i}{[OAA] + K_i}$$
$$(187) \quad f_{SUC} = 0.085\sqrt{[SUC]/[FUM]}$$
$$(188) \quad J_{SDH} = V_{SDH} C2_{inhib} f_{SUC} f_{OAA} \frac{f_Q}{f_Q + K_m}$$
$$(189) \quad J_{c2} = J_{SDH}$$

Parameter	Value	Units	Description
V_{SDH}	0.25	mMHz	Maximum rate of SDH
K_i	0.150	mM	Inhibition constant for oxaloacetate
K_m	0.6	-	Michaelis constant for CoQ

Complex III [3]

$$(190) \quad f_{hi} = [H^+]_i/10^{-7}M$$
$$(191) \quad v_1 = v_Q^{C1} + v_Q^{C2}$$
$$(192) \quad v_2 = k_d([QH_2]_n - [QH_2]_p)$$
$$(193) \quad k_3 = k_{03} K_{eq3} f_{hi}$$
$$(194) \quad k_{-3} = k_{03}$$
$$(195) \quad v_3 = k_3[QH_2]_p[FeS]_{ox} - k_{-3}[Q^{\cdot -}]_p[FeS]_{rd}$$
$$(196) \quad k_{4,ox} = k_{04} K_{eq4,ox} \exp(-\alpha\delta_1\Delta\Psi_m/V_T)$$
$$(197) \quad k_{4,rd} = k_{04} K_{eq4,rd} \exp(-\alpha\delta_1\Delta\Psi_m/V_T)$$
$$(198) \quad k_{-4,ox} = k_{04} \exp(\alpha(1-\delta_1)\Delta\Psi_m/V_T)$$
$$(199) \quad k_{-4,rd} = k_{04} \exp(\alpha(1-\delta_1)\Delta\Psi_m/V_T)$$
$$(200) \quad v_{4,ox} = k_{4,ox}[Q^{\cdot -}]_p[b1] - k_{-4,ox}[Q]_p[b2]$$
$$(201) \quad v_{4,rd} = k_{4,rd}[Q^{\cdot -}]_p[b3] - k_{-4,rd}[Q]_p[b4]$$
$$(202) \quad v_5 = k_d([Q]_p - [Q]_n)$$
$$(203) \quad k_6 = K_{06} K_{eq6} \exp(-\beta\delta_2\Delta\Psi_m/V_T)$$

(204) $k_{-6} = k_{06}\exp(\beta(1-\delta_2)\Delta\Psi_m/V_T)$

(205) $v_6 = k_6[b2] - k_{-6}[b3]$

(206) $k_{7,ox} = k_{07,ox}K_{eq7,ox}\exp(-\gamma\delta_3\Delta\Psi_m/V_T)$

(207) $k_{7,rd} = k_{07,rd}K_{eq7,rd}\exp(-\gamma\delta_3\Delta\Psi_m/V_T)$

(208) $k_{-7,ox} = k_{07,ox}\exp(\gamma(1-\delta_3)\Delta\Psi_m/V_T)$

(209) $k_{-7,rd} = k_{07,rd}\exp(\gamma(1-\delta_3)\Delta\Psi_m/V_T)$

(210) $v_{7,ox} = (k_{7,ox}[Q]_n[b3] - k_{-7,ox}[Q^{\cdot-}]_n[b1])C3_{inhib}$

(211) $v_{7,rd} = (k_{7,rd}[Q]_n[b4] - k_{-7,rd}[Q^{\cdot-}]_n[b2])C3_{inhib}$

(212) $f_{hm} = [H^+]_m/10^{-7}M$

(213) $k_{8,ox} = k_{08,ox}K_{eq8,ox}\exp(-\gamma\delta_3\Delta\Psi_m/V_T)(f_{hm})^2$

(214) $k_{8,rd} = k_{08,rd}K_{eq8,rd}\exp(-\gamma\delta_3\Delta\Psi_m/V_T)(f_{hm})^2$

(215) $k_{-8,ox} = k_{08,ox}\exp(\gamma(1-\delta_3)\Delta\Psi_m/V_T)$

(216) $k_{-8,rd} = k_{08,rd}\exp(\gamma(1-\delta_3)\Delta\Psi_m/V_T)$

(217) $v_{8,ox} = (k_{8,ox}[Q^{\cdot-}]_n[b3] - k_{-8,ox}[QH_2]_n[b1])C3_{inhib}$

(218) $v_{8,rd} = (k_{8,rd}[Q^{\cdot-}]_n[b4] - k_{-8,rd}[QH_2]_n[b2])C3_{inhib}$

(219) $k_9 = k_{09}K_{eq9}$

(220) $k_{-9} = k_{09}$

(221) $v_9 = k_9[FeS]_{rd}[cytc1]_{ox} - k_{-9}[FeS]_{ox}[cytc1]_{rd}$

(222) $k_{10} = k_{010}K_{eq10}$

(223) $k_{-10} = k_{010}$

(224) $v_{10} = k_{10}[Q^{\cdot-}]_p[O_2] - k_{-10}[Q]_p[O_2^{\cdot-}]$

(225) $v_{10b} = v_{10}$

(226) $v_{33} = k_{33}(K_{eq}[cytc1]_{rd}[cytc]_{ox} - [cytc]_{rd}[cytc1]_{ox})$

(227) $\rho'_{C3} = \rho_{C3} \cdot mt_{prot}$

(228) $\rho'_{C4} = \rho_{C4} \cdot mt_{prot}$

(229) $FeS_{rd} = \rho'_{C3} - FeS_{ox}$

(230) $cytc1_{rd} = \rho'_{C3} - cytc1_{ox}$

(231) $cytc_{rd} = \rho'_{C4} - cytc_{ox}$

(232) $[b4] = \rho'_{C3} - [b1] - [b2] - [b3]$

(233) $[QH_2]_p = \Sigma[Q] - [Q]_n - [Q]_p - [QH_2]_n - [Q^{\cdot-}]_p - [Q^{\cdot-}]_n$

(234) $J^{C3}_{hRes} = 2v_3$

(235) $J^{C3}_{ROS,m} = v_{10}$

(236) $J^{C3}_{ROS,i} = v_{10b}$

(237) $\frac{d[Q]_n}{dt} = v_5 - v_{7,ox} - v_{7,rd} - v_1$

(238) $\frac{d[Q^{\cdot-}]_n}{dt} = v_{7,ox} + v_{7,rd} - v_{8,ox} - v_{8,rd}$

(239) $\frac{d[QH_2]_n}{dt} = v_{8,ox} + v_{8,rd} + v_1 - v_2$

(240) $\frac{d[QH_2]_p}{dt} = v_2 - v_3$

(241) $\frac{d[Q^{\cdot-}]_p}{dt} = v_3 - v_{10} - v_{10b} - v_{4,ox} - v_{4,rd}$

(242) $\frac{d[Q]_p}{dt} = v_{10} + v_{10b} + v_{4,ox} + v_{4,rd} - v_5$

(243) $\frac{d[b1]}{dt} = v_{7,ox} + v_{8,ox} - v_{4,ox}$

(244) $\frac{d[b2]}{dt} = v_{4,ox} + v_{7,rd} - v_{8,rd} - v_6$

$$(245)\quad \frac{d[b3]}{dt} \qquad \frac{d[b3]}{dt}$$

$$(246)\quad \frac{d[b4]}{dt} = v_{4,rd} - v_{7,rd} - v_{8,rd}$$

$$(247)\quad \frac{d[FeS]_{ox}}{dt} = v_9 - v_3$$

$$(248)\quad \frac{d[cytc1]_{ox}}{dt} = v_{33} - v_9$$

$$(249)\quad \frac{d[cytc]_{ox}}{dt} = V_e - v_{33}$$

Parameter	Value	Unit	Description
k_{03}	1,666.63	Hz/mM	Reverse rate constant for reaction 3
K_{eq3}	0.6877	-	Equilibrium constant for reaction 3
k_{04}	60.67	Hz/mM	Reverse rate constant for reaction 4
$K_{eq4,ox}$	129.9853	-	Equilibrium constant for reaction 4 (bH oxidized)
$K_{eq4,rd}$	13.7484	-	Equilibrium constant for reaction 4 (bH reduced)
δ_1	0.5	-	
α	0.2497	-	
k_d	22000	Hz	Rate of diffusion across the membrane for Q and QH2
k_{06}	166.67	Hz/mM	Reverse rate constant for reaction 6
K_{eq6}	9.4596	-	Equilibrium constant for reaction 6
δ_2	0.5	-	
β	0.5006	-	
$k_{07,ox}$	13.33	Hz/mM	Reverse rate constant for reaction 7 (bL oxidized)
$K_{eq7,ox}$	3.0748	-	Equilibrium constant for reaction 7 (bL oxidized)
$k_{07,rd}$	1.667	Hz/mM	Reverse rate constant for reaction 7 (bL reduced)

$K_{eq7,rd}$	29.0714	-	Equilibrium constant for reaction 7 (bL reduced)
δ_3	0.5	-	
γ	0.2497	-	$\alpha+\beta+\gamma=1$
$k_{08,ox}$	83.33	Hz/mM	Reverse rate constant for reaction 8 (bL oxidized)
$K_{eq8,ox}$	129.9853	-	Equilibrium constant for reaction 8 (bL oxidized)
$k_{08,rd}$	8.333	Hz/mM	Reverse rate constant for reaction 8 (bL reduced)
$K_{eq8,rd}$	9.4596	-	Equilibrium constant for reaction 8 (bL reduced)
k_{09}	833	Hz/mM	Reverse rate constant for reaction 9
K_{eq9}	0.2697	-	Equilibrium constant for reaction 9
k_{010}	0.8333	Hz/mM	Reverse rate constant for reaction 10
K_{eq10}	1.4541	-	Equilibrium constant for reaction 10
k_{33}	2469.13	Hz/mM	Reverse rate constant for reaction 33
K_{eq33}	2.1145	-	Equilibrium constant for reaction 33
ρ_{C3}	0.325	mM	Total complex III protein

Complex IV [3]

(250) $f_{H_m} = \exp(-\delta_5 \Delta\Psi_m/V_T)([H^+]_m/10^{-7}M)$

(251) $f_{H_i} = \exp((1-\delta_5)\Delta\Psi_m/V_T)([H^+]_i/10^{-7}M)$

(252) $f_{C_{rd}} = [cytc]_{rd}$

(253) $f_{C_{ox}} = \exp((1-\delta_5)\Delta\Psi_m/V_T)[cytc]_{ox}$

(254) $a_{12} = k_{34} f_{C_{rd}}^3 f_{H_m}^4$

(255) $a_{14} = k_{-37} f_{H_i}$

(256) $a_{21} = k_{-34} f_{C_{ox}}^3 f_{H_i}$

(257) $a_{23} = k_{35}[O_2]C4_{inhib}$

(258) $a_{34} = k_{36} f_{C_{rd}} f_{H_m}^3$

$$
\begin{aligned}
&(259) & a_{41} &= k_{37} f_{H_m} \\
&(260) & a_{43} &= k_{-36} f_{C_{ox}} f_{H_i}^2 \\
&(261) & e_1 &= a_{21}a_{41}a_{34} + a_{41}a_{34}a_{23} \\
&(262) & e_2 &= a_{12}a_{41}a_{34} \\
&(263) & e_3 &= a_{23}a_{12}a_{41} + a_{43}a_{14}a_{21} + a_{23}a_{43}a_{12} + a_{23}a_{43}a_{14} \\
&(264) & e_4 &= a_{14}a_{34}a_{21} + a_{34}a_{23}a_{12} + a_{34}a_{23}a_{14} \\
&(265) & \Delta &= e_1 + e_2 + e_3 + e_4 \\
&(266) & Y &= e_1/\Delta \\
&(267) & Yr &= e_2/\Delta \\
&(268) & YO &= e_3/\Delta \\
&(269) & YOH &= e_4/\Delta \\
&(270) & v_{34} &= \rho'_{C4}(Y \cdot a_{12} - Yr \cdot a_{21}) \\
&(271) & v_{35} &= \rho'_{C4} Yr \cdot a_{23} \\
&(272) & v_{36} &= \rho'_{C4}(YO \cdot a_{34} - YOH \cdot a_{43}) \\
&(273) & v_{37} &= \rho'_{C4}(YOH \cdot a_{41} - Y \cdot a_{14}) \\
&(274) & V_e &= 3v_{34} + v_{35} \\
&(275) & J_{hRes}^{C4} &= v_{34} + 2v_{36} + v_{37} \\
&(276) & J_{O_2} &= v_{35} \\
&(277) & J_{hRes} &= J_{hRes}^{C1} + J_{hRes}^{C3} + J_{hRes}^{C4} \\
&(278) & \rho'_{C4} &= \rho_{C4} \cdot mt_{prot}
\end{aligned}
$$

**Parameter**	**Value**	**Unit**	**Description**
$\Sigma cytc$	0.325	mM	Cytochrome c pool
ρ_{C4}	0.325	mM	Complex IV concentration
k_{34}	2.9445E10	Hz/mM^3	@ pH = 7
k_{-34}	290.03	Hz/mM^3	@ pH = 7
k_{35}	45000	Hz/mM	
k_{36}	4.826E11	Hz/mM	@ pH = 7
k_{-36}	4.826	Hz/mM	@ pH = 7
k_{37}	1.7245E8	Hz	@ pH = 7
k_{-37}	17.542	Hz	@ pH = 7

Complex V (ATP synthase) rates [9]

$$
\begin{aligned}
&(279) & J_{F1Fo} &= -\rho^{F1}((100p_a + p_{c1}v_B)v_a - (p_a + p_{c2}v_a)v_h)/\Delta \\
&(280) & J_H^{F1Fo} &= -3\rho^{F1}(100p_a(1 + v_a) - (p_a + p_b)v_h)/\Delta \\
&(281) & \Delta &= (1 + p_1 v_a)v_B + (p_2 + p_3 v_a)v_h \\
&(282) & v_B &= \exp(3\Delta\Psi_B/V_T) \\
&(283) & v_h &= \exp(3\Delta p/V_T)
\end{aligned}
$$

$$(284) \quad v_a = \frac{K'_{eq} \cdot \Sigma[ATP]_m}{\Sigma[Pi]_m \cdot \Sigma[ADP]_m}$$

$$(285) \quad K_{eq}' \quad K_{eqF1}[H^+]_m P_{ATP} P_{H_2O}/(P_{ADP} P_{Pi});$$

Parameter	Value	Unit	Description
ρ_{F1}	5	mM	Concentration of F1-Fo ATPase
K'_{eq}	6.47E5	M	Apparent equilibrium constant for ATP hydrolysis From Golding's work [10]
K_{eqF1}	1.71E6		
$\Delta\Psi_B$	50	mV	Phase boundary potential
p_a	1.656E-5	Hz	Sum of products of rate constants
p_b	3.373E-7	Hz	Sum of products of rate constants
p_{c1}	9.651E-14	Hz	Sum of products of rate constants
p_{c2}	4.585E-14	Hz	Sum of products of rate constants
p_1	1.346E-4	-	Sum of products of rate constants
p_2	7.739E-7	-	Sum of products of rate constants
p_3	6.65E-15	-	Sum of products of rate constants

Reactive oxygen species (ROS) scavenging and transport were calculated as follows:

Catalase (CAT) [11] includes inhibition by high levels of hydrogen peroxide

$$(286) \quad V_{CAT} = 2k_1 E_T [\mathrm{H_2O_2}]_i \cdot e^{-fr[\mathrm{H_2O_2}]_i}$$

Parameter	Value	Unit	Description
k_1	17000	mM*Hz	Rate constant
E_T	1	nM	Extra-matrix concentration of catalase
fr	0.05	1/mM	Hydrogen peroxide inhibition factor

Superoxide dismutase (SOD) [11] is based on McADAM, 1976 model, for both cytosolic and mitochondrial compartments.

$$(287)\quad J_{SOD} = \frac{2k_5 E_T f_{sox}(k_1 + k_3')}{k_5(2k_1 + k_3') + k_3' f_{sox}}$$

$$(288)\quad k_3' = k_3(1 + \frac{[\mathrm{H_2O_2}]}{K_{H_2O_2}})$$

$$(289)\quad f_{sox} = k_1^{SOD}[\mathrm{O_2}^-]$$

Parameter	Value	Unit	Description
k_1	1200000	Hz/mM	Rate constant for EA -> EB
k_3	24000	Hz/mM	Rate constant for EB -> EC
k_5	0.24	Hz	Rate constant for EC -> EA
K_i	0.5	mM	Inhibition constant for H2O2
$E_{T,i}$	0.0003	mM	Concentration of Cu,ZnSOD (cytosolic)
$E_{T,m}$	0.00024	mM	Concentration of MnSOD (mitochondrial)

Glutathione (GSH) systems [11] were calculated as follows

Glutathione peroxidase (GPX) uses Dalziel type Ping-pong mechanism, for both cytosolic and mitochondrial compartments.

$$(290)\quad J_{GPX} = \frac{E_T}{A + B}$$

$$(291)\quad A = \frac{\Phi_1}{[\mathrm{H_2O_2}]}$$

$$(292)\quad B = \frac{\Phi_2}{[\mathrm{GSH}]}$$

Parameter	Value	Unit	Description
$E_{T,i}$	50	nM	GPX content (cytosolic)
$E_{T,m}$	50	nM	GPX content (mitochondrial)
Φ_1	5E-6	mM*s	Dalziel coefficient
Φ_2	7.5-E4	mM*s	Dalziel coefficient

Glutathione reductase (GR) follows Michaelis-Menten kinetics, for both cytosolic and mitochondrial compartments.

$$(293)\quad J_{GR} = k_1 E_T \frac{A}{1+A}\frac{B}{1+B}$$
$$(294)\quad A = \frac{[\mathrm{GSSG}]}{K_{GSSG}}$$
$$(295)\quad B = \frac{[\mathrm{NADPH}]}{K_{NADPH}}$$

Parameter	Value	Unit	Description
$E_{T,i}$	9E-4	mM	GR content (cytosolic)
$E_{T,m}$	9E-4	mM	GR content (mitochondrial)
k_1	2.5	Hz	Catalytic constant of GR
K_{GSSG}	0.06	mM	Michaelis constant for GSSG
K_{NADPH}	0.015	mM	Michaelis constant for NADPH

Glutaredoxin system [12] (disabled in the cellular model)

$$(296)\quad J_{GRX} = V_{max} BC$$
$$(297)\quad B = \frac{[\mathrm{PSSG}]}{[\mathrm{PSSG}] + K_{PSSG}}$$
$$(298)\quad C = \frac{A\Sigma[\mathrm{Grx}]}{A\Sigma[\mathrm{Grx}] + K_m}$$
$$(299)\quad A = \frac{K_{eq}[\mathrm{GSH}]^2}{K_{eq}[\mathrm{GSH}]^2 + [\mathrm{GSSG}]}$$

Parameter	Value	Unit	Description
$V_{max,i}$	3.6E-4	mM*Hz	Extra-matrix glutaredoxin reaction rate
$V_{max,m}$	3.6E-4	mM*Hz	Mitochondrial glutaredoxin reaction rate
K_{eq}	1.37E-3	1/mM	Equilibrium constant of glutaredoxin
K_m	0.01	mM	Michaelis constant for GSH of GRX
K_{PSSG}	0.0005	mM	Michaelis constant for glutathionylated protein of glutaredoxin
$\Sigma[\mathrm{Grx}]$	0.002	mM	Glutaredoxin concentration
$V_{max,i}$	0		Cellular model

$V_{max,i}$	0		Cellular model

Glutathionylated protein [12] (disabled in the cellular model)

$$(300)\quad J_{PSSG} = k_1 E_T (\Sigma[\mathrm{PSSG}] - [\mathrm{PSSG}]) AB$$

$$(301)\quad A = \frac{[\mathrm{GSH}]}{[\mathrm{GSH}] + K_m}$$

$$(302)\quad B = \frac{K_{act}}{[\mathrm{H_2O_2}] + K_{act}}$$

Parameter	Value	Unit	Description
k_1	640	Hz	Rate constant of protein glutathionylation
E_T	8E-4		Concentration of proteins that can become glutathionylated
$\Sigma[\mathrm{PSSG}]$	1E-3	mM	Total PSSG
K_m	0.75	mM	Michaelis constant of GSH
K_{act}	1E-3	mM	Activation constant of H2O2
k_1	0		Cellular model

Glutathione transport [12] (disabled in the cellular model)

$$(303)\ J_{GST} = c_{GST} \frac{[\mathrm{GSH}]_i - [\mathrm{GSH}]_m}{[\mathrm{GSH}]_i + k_{0.5}}$$

Parameter	Value	Unit	Description
c_{GST}	1.5E-5	mM * Hz	Rate constant of glutathione transporter
$k_{0.5}$	2.6	mM	Transport association constant of GSH
c_{GST}	0		Cellular model

Conservation relationship of glutathione for both cytosolic and mitochondrial compartments.

$$(304)\ \Sigma[\mathrm{GSH}] = [\mathrm{GSH}] + 2[\mathrm{GSSG}]$$

Parameter	Value	Unit	Description
$[\mathrm{GSH}]_i$	1.65	mM	Cytosolic GSH pool

$[\mathrm{GSH}]_m$	1.65	mM	Mitochondrial GSH pool

Thioredoxin system [12] was calculated as follows:

Peroxiredoxin (TPX) uses Dalziel type Ping-pong mechanism for both cytosolic and mitochondrial compartments.

$$(305)\quad J_{GPX} = \frac{E_T}{A+B}$$

$$(306)\quad A = \frac{\Phi_1}{[\mathrm{H_2O_2}]}$$

$$(307)\quad B = \frac{\Phi_2}{[\mathrm{TrxSH_2}]}$$

Parameter	Value	Unit	Description
$E_{T,i}$	100	µM	GPX content (cytosolic)
$E_{T,m}$	3	µM	GPX content (mitochondrial)
Φ_1	3.83	mM * s	Dalziel coefficient
Φ_2	1.85	mM * s	Dalziel coefficient

Thioredoxin reductase (TR) follows Michaelis-Menten kinetics, for both cytosolic and mitochondrial compartments.

$$(308)\quad J_{TR} = k_1 E_T \frac{A}{1+A}\frac{B}{1+B}$$

$$(309)\quad A = \frac{[\mathrm{TrxSS}]}{K_{TrxSS}}$$

$$(310)\quad B = \frac{[\mathrm{NADPH}]}{K_{NADPH}}$$

Parameter	Value	Unit	Description
$E_{T,i}$	0.35	µM	TR content (cytosolic)
$E_{T,m}$	0.35	µM	TR content (mitochondrial)
k_1	22.75	Hz	Catalytic constant of TR
K_{TrxSS}	35	µM	Michaelis constant for TrxSS
K_{NADPH}	65	µM	Michaelis constant for NADPH

Conservation relationship of thioredoxin for both cytosolic and mitochondrial compartments.

$$(311)\ [\mathrm{TrxSS}] = \Sigma[\mathrm{Trx}] - [\mathrm{TrxSH_2}]$$

Parameter	Value	Unit	Description
$[\mathrm{TrxSS}]_i$	25	μM	Sum of cytosolic thioredoxin
$[\mathrm{TrxSS}]_m$	50	μM	Sum of mitochondrial thioredoxin

Inner mitochondrial anion channel [11] was calculated as follows:

$$\begin{aligned}
(312)\quad g_{IMAC} &= (a + b\frac{[\mathrm{O_2}^-]_\mathrm{i}}{[\mathrm{O_2}^-]_\mathrm{i} + K_{CC}})(G_L + \frac{G_{max}}{1 + e^{\kappa(\Delta\Psi_m^b + \Delta\Psi_m)}}) \\
(313)\quad V_{IMAC} &= g_{IMAC}\Delta\Psi_m \\
(314)\quad V_{tr}^{ROS} &= j \cdot g_{IMAC}(\Delta\Psi_m + V_T ln(\frac{[\mathrm{O_2}^-]_m}{[\mathrm{O_2}^-]_\mathrm{i}}))
\end{aligned}$$

Parameter	Value	Unit	Description
a	0.001	-	Basal IMAC conductance
b	10000	-	Activation factor by
K_{CC}	10	μM	Activation constant by
G_L	0.035	μM * Hz / mV	Integral conductance for IMAC
G_{max}	3.9085	μM * Hz / mV	Leak conductance of IMAC
κ	0.07	1/mV	Steepness factor
$\Delta\Psi_m^b$	4	mV	Potential at half saturation
j	0.1	-	Fraction of IMAC conductance

Hydrogen peroxide transfer [12] was calculated using simple diffusion as follows:

$$(315)\ J_{diff}^{H_2O_2} = c_{diff}([\mathrm{H_2O_2}]_m - [\mathrm{H_2O_2}]_i)$$

Parameter	Value	Unit	Description
c_{diff}	0.2	Hz	Diffusion rate across IMM

Conservation of NADPH was calculated as follows:

$$(316)\ \Sigma[\text{NADP}]_m = [\text{NADP}^+]_m + [\text{NADPH}]_m$$

NADPH-producing isocitrate dehydrogenase (IDH2) [12] was calculated as follows:

$$(317)\quad J_{IDH2} = f_H \frac{V_f AB - V_b PQ}{(1+A+P)(1+B+Q)}$$
$$(318)\quad A = [\text{ISOC}]/K_{m,ISOC}$$
$$(319)\quad B = ([\text{NADP}]_m + K_{i,NADP})/K_{m,NADP}$$
$$(320)\quad P = [\alpha\text{KG}]/K_{m,\alpha KG}$$
$$(321)\quad Q = ([\text{NADPH}]_m/K_{m,NADPH})$$
$$(322)\quad f_H = \frac{K_H}{K_H + [\text{H}^+]_m}$$

Parameter	Value	Unit	Description
K_H	500	µM	Dissociation constant for H
$K_{m,ISOC}$	3.9	µM	Michaelis constant for isocitrate
$K_{m,NADP}$	6.7	µM	Michaelis constant for NADP
$K_{i,NADP}$	0.002	µM	Inhibition constant for NADP
$K_{m,NADPH}$	12	µM	Michaelis constant for NADPH
$K_{m,\alpha KG}$	510	µM	Michaelis constant for αKG
V_f	87	µM*Hz	Maximal forward rate of IDH2
V_b	5.45	µM*Hz	Maximal backward rate of IDH2

Transhydrogenase (THD) [12] was calculated as follows:

$$(323)\quad J_{THD} = (V_f AB' - V_b P'Q)/\Delta$$
$$(324)\quad \Delta = 1 + A + B + P + Q + AQ + B'P' + AB' + P'Q$$
$$(325)\quad A = [\text{NADH}]/K_{m,NADH}$$
$$(326)\quad B = [\text{NADP}]_m/K_{m,NADP}$$
$$(327)\quad B' = B \cdot \exp(x(d-1)\Delta p/V_T)$$
$$(328)\quad P = [\text{NAD}]/K_{m,NAD}$$
$$(329)\quad P' = P \cdot \exp(xd\Delta p/V_T)$$
$$(330)\quad Q = [\text{NADPH}]_m/K_{m,NADPH}$$
$$(331)\quad V_f = E_T k_a$$
$$(332)\quad V_b = V_f \frac{K_{m,NADP} K_{m,NADH}}{K_{m,NAD} K_{m,NADPH}^{THD} K_{eq}^{App}}$$

Parameter	Value	Unit	Descripton
$K_{m,NADPH}$	20	µM	Michaelis constant for NADPH
$K_{m,NADH}$	10	µM	Michaelis constant for NADH
$K_{m,NAD}$	125	µM	Michaelis constant for NAD
$K_{m,NADP}$	17	µM	Michaelis constant for NADP
E_T	0.01187	µM	Concentration of THD
k_a	1174.74	Hz	Forward catalytic constant
K_{eq}^{App}	1	-	Apparent equilibrium constant

Supplementary References

1. Fu X*, et al.* Specialized fibroblast differentiated states underlie scar formation in the infarcted mouse heart. *J Clin Invest* **128**, 2127-2143 (2018).

2. Kongas O, van Beek J. Creatine kinase in energy metabolic signaling in muscle. *Nature Precedings*, (2007).

3. Gauthier Laura D, Greenstein Joseph L, O'Rourke B, Winslow Raimond L. An Integrated Mitochondrial ROS Production and Scavenging Model: Implications for Heart Failure. *Biophys J* **105**, 2832-2842 (2013).

4. Gupta A*, et al.* Creatine kinase-mediated improvement of function in failing mouse hearts provides causal evidence the failing heart is energy starved. *J Clin Invest* **122**, 291-302 (2012).

5. Harrison GJ, Wijhe MHv, Groot Bd, Dijk FJ, Gustafson LA, Beek JHGMv. Glycolytic buffering affects cardiac bioenergetic signaling and contractile reserve similar to creatine kinase. *American Journal of Physiology-Heart and Circulatory Physiology* **285**, H883-H890 (2003).

6. Hettling H, van Beek JH. Analyzing the functional properties of the creatine kinase system with multiscale 'sloppy' modeling. *PLoS computational biology* **7**, e1002130 (2011).

7. Vinnakota KC, Bassingthwaighte JB. Myocardial density and composition: a basis for calculating intracellular metabolite concentrations. *American Journal of Physiology-Heart and Circulatory Physiology* **286**, H1742-H1749 (2004).

8. Belke DD, Swanson EA, Dillmann WH. Decreased Sarcoplasmic Reticulum Activity and Contractility in Diabetic db/db Mouse Heart. *Diabetes* **53**, 3201-3208 (2004).

9. Wei AC, Aon MA, O'Rourke B, Winslow RL, Cortassa S. Mitochondrial energetics, pH regulation, and ion dynamics: a computational-experimental approach. *Biophys J* **100**, 2894-2903 (2011).

10. Golding EM, Teague WE, Jr., Dobson GP. Adjustment of K' to varying pH and pMg for the creatine kinase, adenylate kinase and ATP hydrolysis equilibria permitting quantitative bioenergetic assessment. *The Journal of experimental biology* **198**, 1775-1782 (1995).

11. Cortassa S, Aon MA, Winslow RL, O'Rourke B. A mitochondrial oscillator dependent on reactive oxygen species. *Biophys J* **87**, 2060-2073 (2004).

12. Kembro JM, Aon MA, Winslow RL, O'Rourke B, Cortassa S. Integrating mitochondrial energetics, redox and ROS metabolic networks: a two-compartment model. *Biophys J* **104**, 332-343 (2013).
