# Supplementary material for: Mitochondrial CaMKII causes adverse metabolic reprogramming and dilated cardiomyopathy
Source: Nat Commun. 2020 Sep 4;11:4416. doi: 10.1038/s41467-020-18165-6 (PMC7473864; doi:10.1038/s41467-020-18165-6)
Supplement: Supplementary file 3 — Description of Additional Supplementary Files [file 41467_2020_18165_MOESM3_ESM.docx]

**Supplementary Movie 1: MR Cine**

Representative cine magnetic resonance images acquired at 4.7T showing left ventricular short-axis cross sections at the level of the papillary muscles during systole in WT, CKmito, mtCaMKII, and mtCaMKII x CKmito interbred mice. The field-of-view is 32mm x 32mm.
