# Supplementary material for: Mitochondrial CaMKII causes adverse metabolic reprogramming and dilated cardiomyopathy
Source: Nat Commun. 2020 Sep 4;11:4416. doi: 10.1038/s41467-020-18165-6 (PMC7473864; doi:10.1038/s41467-020-18165-6)
Supplement: Supplementary file 5 — Reporting Summary [file 41467_2020_18165_MOESM5_ESM.pdf]

Corresponding author(s): Anderson, Mark
Luczak, Elizabeth
Wei, An-Chi

Last updated by author(s): 2020-07-30

Reporting Summary

Nature Research wishes to improve the reproducibility of the work that we publish. This form provides structure for consistency and transparency in reporting. For further information on Nature Research policies, see our Editorial Policies and the Editorial Policy Checklist.

Statistics

For all statistical analyses, confirm that the following items are present in the figure legend, table legend, main text, or Methods section.

n/a	Confirmed	
☐	☒	The exact sample size (n) for each experimental group/condition, given as a discrete number and unit of measurement
☐	☒	A statement on whether measurements were taken from distinct samples or whether the same sample was measured repeatedly
☐	☒	The statistical test(s) used AND whether they are one- or two-sided<br>*Only common tests should be described solely by name; describe more complex techniques in the Methods section.*
☒	☐	A description of all covariates tested
☒	☐	A description of any assumptions or corrections, such as tests of normality and adjustment for multiple comparisons
☐	☒	A full description of the statistical parameters including central tendency (e.g. means) or other basic estimates (e.g. regression coefficient) AND variation (e.g. standard deviation) or associated estimates of uncertainty (e.g. confidence intervals)
☐	☒	For null hypothesis testing, the test statistic (e.g. F, t, r) with confidence intervals, effect sizes, degrees of freedom and P value noted<br>*Give P values as exact values whenever suitable.*
☒	☐	For Bayesian analysis, information on the choice of priors and Markov chain Monte Carlo settings
☒	☐	For hierarchical and complex designs, identification of the appropriate level for tests and full reporting of outcomes
☒	☐	Estimates of effect sizes (e.g. Cohen's d, Pearson's r), indicating how they were calculated

Our web collection on statistics for biologists contains articles on many of the points above.

Software and code

Policy information about availability of computer code

Data collection	Image Studio 4.0 (Licor), Zen (Zeiss), NIS Elements 4.00 (Nikon), Gen5 1.09.8 (BioTek), CFX Manager 3.1 (BioRad), Vevo Lab 3.1.1 (Visualsonics)
Data analysis	Image Studio 4.0 (Licor), ImageJ 1.5, GraphPad Prism 7, Aperio ImageScope 9, Proteome Discoverer (version 1.3, Thermo Scientific), MATLAB 2018b (The MathWorks, Natick, MA), NIS Elements 4.00 (Nikon), Vevo Lab 3.1.1 (Visualsonics), CFX Manager 3.1 (BioRad)

For manuscripts utilizing custom algorithms or software that are central to the research but not yet described in published literature, software must be made available to editors and reviewers. We strongly encourage code deposition in a community repository (e.g. GitHub). See the Nature Research guidelines for submitting code & software for further information.

Data

Policy information about availability of data

All manuscripts must include a data availability statement. This statement should provide the following information, where applicable:

- Accession codes, unique identifiers, or web links for publicly available datasets
- A list of figures that have associated raw data
- A description of any restrictions on data availability

The authors declare that the data supporting the findings of this study are available within the paper and its supplementary information files. Source data are provided with this paper. The mass spectrometry proteomics data are available via the ProteomeXchange Consortium via the PRIDE 109 partner repository with the dataset identifier PXD004631 [http://proteomecentral.proteomexchange.org/cgi/GetDataset?ID=PXD004631]. The data that support the findings of this study and unique materials are available from the corresponding authors upon reasonable request.

Field-specific reporting

Please select the one below that is the best fit for your research. If you are not sure, read the appropriate sections before making your selection.

☒ Life sciences ☐ Behavioural & social sciences ☐ Ecological, evolutionary & environmental sciences

For a reference copy of the document with all sections, see nature.com/documents/nr-reporting-summary-flat.pdf

Life sciences study design

All studies must disclose on these points even when the disclosure is negative.

Sample size	No statistical test was used to predetermine sample size. Sample size was chosen based on our prior studies using the same types of assays, as well as published literature (Joiner et al [25], Gupta et al [55], Rasmussen et al [82], Kovanich et al [111]), to ensure statistically significant results.
Data exclusions	No data was excluded from analysis.
Replication	All animal studies were performed with animals from multiple litters over time. Western blots, gels, qPCR, and biochemical assays were performed at least twice independently. Proteomics was performed once. All attempts at replication were successful.
Randomization	All animals and cells were randomly assigned to the experimental groups.
Blinding	Investigators were blinded to groups during data collection and analysis.

Reporting for specific materials, systems and methods

We require information from authors about some types of materials, experimental systems and methods used in many studies. Here, indicate whether each material, system or method listed is relevant to your study. If you are not sure if a list item applies to your research, read the appropriate section before selecting a response.

Materials & experimental systems

n/a	Involved in the study	
☐	☒	Antibodies
☒	☐	Eukaryotic cell lines
☒	☐	Palaeontology and archaeology
☐	☒	Animals and other organisms
☒	☐	Human research participants
☒	☐	Clinical data
☒	☐	Dual use research of concern

Methods

n/a	Involved in the study	
☒	☐	ChIP-seq
☒	☐	Flow cytometry
☒	☐	MRI-based neuroimaging

Antibodies

Antibodies used	CaMKII (abcam ab181052), phospho-CaMKII (Thermo Scientific MA1-047), VDAC1 (abcam ab14734, FLAG (Rockland 600-401-383), GAPDH (Cell Signaling 5174), CK mito (Sigma C2244), CoxIV (Cell Signaling 4844), CK-M (Sigma C1869), α-actinin (Sigma A7811), OxPhos (abcam ab110413), AcLys (Cell Signaing 9814), HA (Sigma H3663), SERCA2a (Badrilla A010-20), PDH (abcam ab110330), CD45 (Thermo 53-0452-82), Goat anti-Rat Alexafluor 555 (Thermo A21434)
Validation	CaMKII see https://www.abcam.com/camkii-delta-antibody-epr13095-ab181052.html#description_images_4; phospho-CaMKII see https://www.thermofisher.com/antibody/product/Phospho-CaMKII-alpha-Thr286-Antibody-clone-22B1-Monoclonal/MA1-047; VDAC1 see https://www.abcam.com/vdac1-porin-antibody-20b12af2-ab14734.html#description_images_1; FLAG see https://rockland-inc.com/store/Antibodies-to-FLAG-and-Antibodies-to-6XHIS-Tags-600-401-383-O4L_23854.aspx; GAPDH see https://www.cellsignal.com/products/primary-antibodies/gapdh-d16h11-xp-rabbit-mab/5174;CK mito see https://www.sigmaaldrich.com/catalog/product/sigma/c2244?lang=en&region=US and Supplementary Figure 5a; CoxIV see https://www.cellsignal.com/products/primary-antibodies/cox-iv-antibody/4844; CK-M see https://www.sigmaaldrich.com/catalog/product/sigma/c1869?lang=en&region=US and Supplementary Figure 5a; α-actinin see https://www.sigmaaldrich.com/catalog/product/sigma/a7811?lang=en&region=US;<br>OxPhos (Abcam, ab110413, 1:500), https://www.abcam.com/total-oxphos-rodent-wb-antibody-cocktail-ab110413.html#description_images_3; AcLys see https://www.cellsignal.com/products/primary-antibodies/acetylated-lysine-ack-2-100-multimab-rabbit-mab-mix/9814; HA see https://www.sigmaaldrich.com/catalog/product/sigma/h3663?lang=en&region=US; SERCA2a see https://badrilla.com/serca2a-pab-serum.html; PDH see https://www.abcam.com/pyruvate-dehydrogenase-e1-alpha-subunit-antibody-9h9af5-ab110330.html; CD45 see https://www.thermofisher.com/antibody/product/CD45R-B220-Antibody-clone-RA3-6B2-Monoclonal/53-0452-82

Animals and other organisms

Policy information about studies involving animals; ARRIVE guidelines recommended for reporting animal research

Laboratory animals	Mice used in these studies were a mixture of male and female animals 7-20 weeks of age unless otherwise noted in the figure legend. Mice were bred on the C57Bl/6J or CD1 background. Genetic mouse models used include: mtCaMKIIN, mtCaMKII, AC3-I, CKmito, aMHC-Tet-off, mCat, Ppif-/-. New models are described in the methods section, and all previously published models are referenced in the manuscript text.
Wild animals	This study did not involve wild animals.
Field-collected samples	This study did not involve samples collected from the field.
Ethics oversight	All the experiments were carried out in accordance with the guidelines and approval of Institutional Animal Care and Use Committees at the University of Iowa and Johns Hopkins University. (PHS Animal Welfare Assurance, A3021-01 (Univ. Iowa), A3272-01 (JHU)).

Note that full information on the approval of the study protocol must also be provided in the manuscript.
